# Isocorydine Derivatives and Their Anticancer Activities

**DOI:** 10.3390/molecules190812099

**Published:** 2014-08-12

**Authors:** Mei Zhong, Yanjuan Liu, Junxi Liu, Duolong Di, Mengrou Xu, Yaya Yang, Wenguang Li, Yali Chen, Jinxia Liu

**Affiliations:** 1 Key Laboratory of Chemistry of Northwestern Plant Resources and Key Laboratory for Natural Medicine of Gansu Province, Lanzhou Institute of Chemical Physics, Chinese Academy of Sciences, Lanzhou 730000, China; E-Mails: zhongmei@licp.cas.cn (M.Z.); liuyanjuan09@163.com (Y.L.); didl@licp.cas.cn (D.D.); 2 Graduate University of the Chinese Academy of Sciences, Beijing 100049, China; 3 Gansu Key Laboratory of Preclinical Study for New Drugs, Institute of Pharmacology, School of Basic Medical Science, Lanzhou University, Lanzhou 730000, China; E-Mails: xmr1991@126.com (M.X.); yangmegan2011@hotmail.com (Y.Y.); liwg@lzu.edu.cn (W.L.); chenyl2013@licp.cas.cn (Y.C.); 4 Institute of Biology, Gansu Academy of Sciences, Lanzhou 730000, China; E-Mail: Liujinx0168@163.com

**Keywords:** aporphine alkaloids, isocorydine, synthesis, anticancer activity

## Abstract

In order to improve the anticancer activity of isocorydine (ICD), ten isocorydine derivatives were prepared through chemical structure modifications, and their *in vitro* and *in vivo* activities were experimentally investigated. 8-Amino-isocorydine (**8**) and 6a,7-dihydrogen-isocorydione (**10**) could inhibit the growth of human lung (A549), gastric (SGC7901) and liver (HepG2) cancer cell lines *in vitro*. Isocorydione (**2**) could inhibit the tumor growth of murine sarcoma S_180_-bearing mice, and 8-acetamino-isocorydine (**11**), a pro-drug of 8-amino-isocorydine (**8**), which is instable in water solution at room temperature, had a good inhibitory effect on murine hepatoma H_22_-induced tumors. The results suggested that the isocorydine structural modifications at C-8 could significantly improve the biological activity of this alkaloid, indicating its suitability as a lead compound in the development of an effective anticancer agent.

## 1. Introduction

Aporphine alkaloids belong to benzylisoquinoline alkaloids, existing in 20 families and more than 100 genera of plants. These alkaloids share the common characteristic of a tetracyclic skeleton, albeit with different substituents. The aporphine template is associated with a wide range of biological activities, such as acting as a dopaminergic agent, anti-platelet activities, anti-oxidative properties and cytotoxic activities. Because of their attractive biological activities, many studies have focused on the potential of aporphinoid alkaloids in drug development, and the anticancer activity of these compounds has become a hot pharmaceutical research area in recent years [1,2,3,4,5,6]. Isocorydine (**1**), an aporphine alkaloid, is widely present in many plants, including *Dicranostigma leptopodum* (Maxim) Fedde, which is mainly distributed in the northwest of China. In our continued investigation to find antitumor compounds from natural resources, we found that isocorydine was abundant in this plant [7,8]. Recent research has demonstrated that isocorydine not only inhibits cell proliferation in hepatocellular carcinoma cell lines by inducing G2/M cell cycle arrest and apoptosis, but also targets the drug-resistant cellular side population (or cancer stem cells) through inducing PDCD4-related apoptosis. Furthermore, isocorydine could selectively reduce the size and weight of the side population cell-induced tumor masses in nude mice, which suggested that isocorydine is a potential therapeutic drug for targeting the side population cancer cells of hepatocellular carcinoma [9,10]. Cancer stem cells show self-renewal properties and chemoresistance to the majority of anticancer agents, which is a challenge in clinical chemotherapy. Isocorydine could not only significantly reduce the percentage of CD133^+^ and EpCAM-expressing cells, two types of cancer stem cells, but also could suppress the ability of primary liver carcinoma PLC/PRF/5 CD133^+^ cells to form hepatospheres and tumor-like spheres *in vitro* [9,10,11]. However, isocorydine shows only intermediate antitumor ability, and the effective dosage could reach up 200 μM, which is a relatively high dosage for clinical treatment [10]. In order to reduce the dosage of isocorydine needed to achieve an effective outcome and improve the anticancer activity, we used chemical modification and structure transformation to obtain a series of isocorydine derivatives. We here report the results of bioactivity investigations and summarize the primary structure-activity relationships of isocorydine derivatives as antitumor agents.

## 2. Results and Discussion

### 2.1. Chemistry

**Figure 1:**
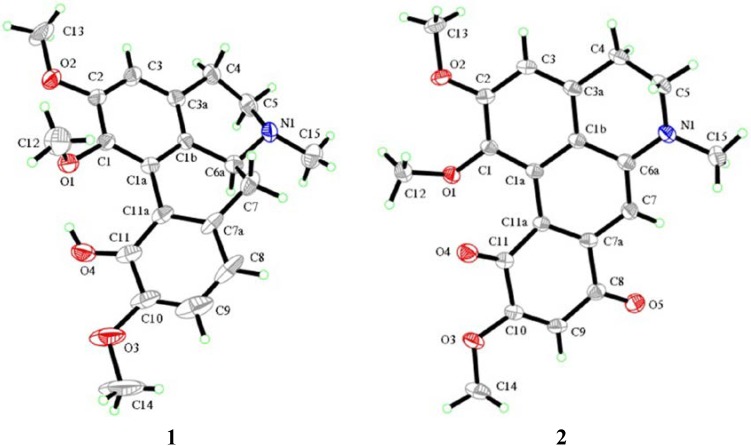
X-ray crystal structures of **1** and **2**. The molecular planarity of **2** was superior to that of **1** due to the double bond of C-6a and C-7, which could conjugate with benzene rings in **2** and reduce the dihedral angle of biphenyl to zero. The chirality of C-6a in **1** resulted in non-rigid conformation of the ring of B and C.

**Scheme 1:**
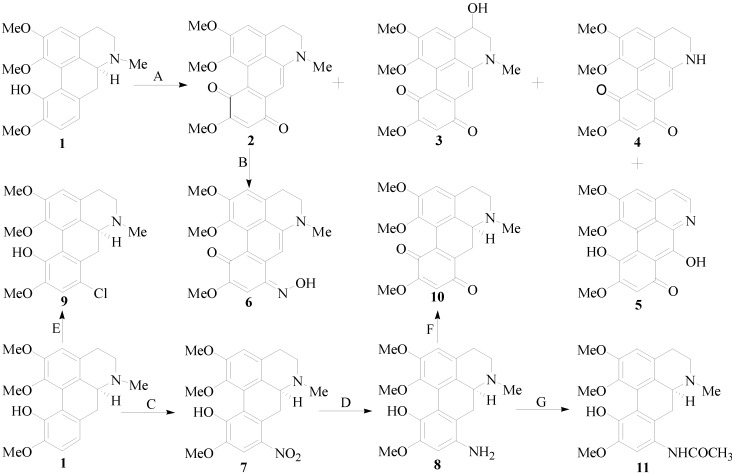
The synthetic route of isocorydine derivatives. *Reagents and conditions*: (**A**) ON(SO_3_K)_2_/H_2_O/Na_2_HPO_4_, 25 °C, stir, 24.0 h; (**B**) NH_2_OH·HCl/EtOH, 80 °C, reflux, 1.5 h; (**C**) HNO_3_/H_2_SO_4_/CHCl_3_/CH_2_Cl_2_, −30 °C, stir, 1.5 h; (**D**) 0.3 MPa H_2_, Pd/C, EtOH, 25 °C, stir, 2.5 h; (**E**) NCS/CH_3_COOH, 7 °C, stir, 2.0 h; (**F**) NaNO_2_/HCl, 0 °C, stir, 1.0 h; (**G**) CH_3_COCl, stir, 2.0 h.

The synthetic routes of isocorydine derivatives are shown in Scheme 1. Modifications at the C-8 position in the D ring of isocorydine were the focus of our work. Ten aporphine compounds were obtained through structural modifications of isocorydine. The starting material, isocorydine (**1**) as a colorless crystal, was isolated from *D. leptopodum* (Maxim) Fedde using column chromatography on a silica gel in our laboratory. Its ^1^H, ^13^C nuclear magnetic resonance (NMR) spectra and its X-ray crystal structure (Figure 1) were consistent with data reported in former literature [12,13]. While isolating the chemical constituents of *D. leptopodum* (Maxim) Fedde, a small amount of isocorydione (**2**), which has a p-benzoquinonyl structure, was also isolated. However, the low content of **2** in the plant limited the ability to screen its activity in the pharmacology experiments. Chia *et al.* successfully obtained norfissilandione, which retained the p-benzoquinonyl segment through fissoldine oxidized by Fremy’s radical, which is a mild oxidant and can transform the phenolic hydroxyl segment to p-benzoquinonyl [14]. Using this method, Compound **2** was synthesized through oxidization of **1** by Fremy’s radical, and Compounds **3**, **4** and **5** were also isolated as by-products of this reaction. The oxidization mechanism of isocorydine involved four molecular of Fremy’s radicals, which resulted in isocorydine losing relevant hydrogen atoms in its chemical structure. The hydrogen atom located at C-8 possesses high chemical reactivity and is easily lost. Considering the low polarity of **2**, Compound **6** was prepared through the nucleophilic addition reaction between hydroxylamine hydrochloride with the carbonyl group at position C-8 of **2**. Nitration of isocorydine at −30 °C generated **7**, which contained a nitro-group at C-8. The low temperature was necessary in this experiment, as oxidation of the phenolic hydroxyl in isocorydine can easily occur by reaction with nitric acid. Due to the steric hindrance of the methoxyl group at C-2, no nitro-substituted product was obtained at C-3. In order to conduct a relatively clean reaction involving green chemistry, **7** was reduced under hydrogen pressure at 0.3 MPa and was catalyzed by palladium/carbon hydrogenation catalyst (10%) to obtain **8**, which is a brown amorphous powder. The reduction reaction required neutral mild conditions, for Compound **8** was not stable at room temperature and could be easily degraded. Even the weak oxidant sodium nitrite could oxidize **8** to **10** at 0 °C. Compound **9** was obtained through electrophilic substitution between isocorydine and a chloride atom of N-chlorosuccinimide, a typical chloridizing reagent.

**Figure 2:**
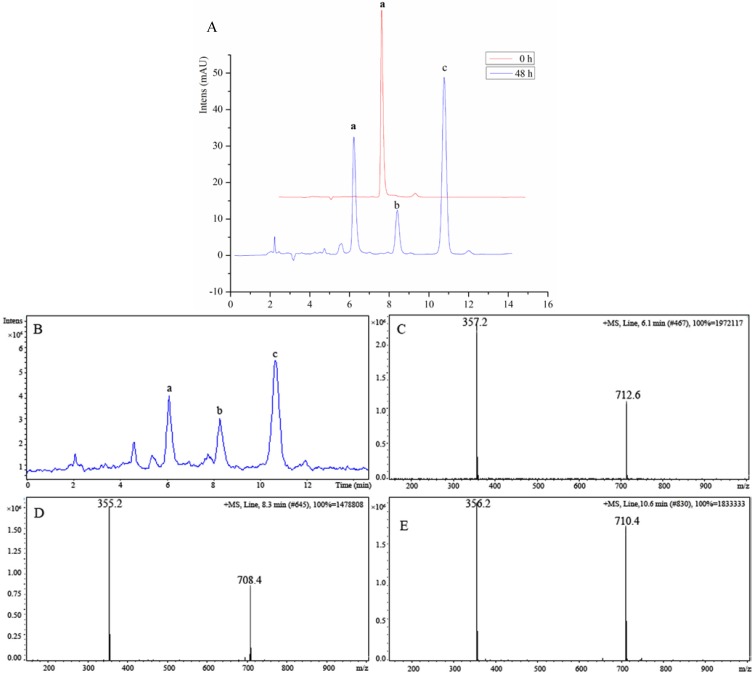
Evaluation of the instability of 8-amino-isocorydine (**8**) using the HPLC-UV-MS method. (**A**) Representative chromatogram of the degradation products of 8-amino-isocorydine (**8**) in water solution at room temperature over 48 h. (a: Compound **8**; b and c are the main degradation products.). The mobile phase conditions were as follows: UV detector set at 270 nm, a SinoCrom ODS-BP C18 column (4.6 × 250 mm, 5 μm) was used with the mobile phase consisting of MeOH: water (65:35, using aqua ammonia adjusted pH at 7.2) at a flow rate of 1.0 mL/min. The column temperature was maintained at 25 °C; (**B**) Total ion chromatogram (TIC) of **8** by the LC-MSD-trap method. MS detection was conducted by ESI and operated in positive mode; (**C**) MS spectra tracked for a, *m/z* = 357.2 at 6.1 min; (**D**) MS spectra tracked for b, *m/z* = 355.2 at 8.3 min; (**E**) MS spectra tracked for c, *m/z* = 357.2 at 10.6 min.

**Scheme 2:**
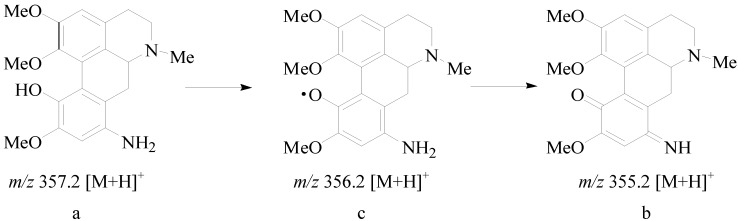
The main degrade products and the degrade route of 8-amino-isocorydine (**8**) in water solution at room temperature.

During mice biological activity experiments, we discovered that the color of the water solution of **8** became darker over time, suggesting that **8** was not stable in water solution. Therefore, the degrading mechanism of **8** was further investigated using the liquid chromatography-mass spectrometry (LC-MS)-trap method. In comparing the chromatogram of **8** at different time points, such as between the time of fresh preparation and after 48 h of standing at room temperature (Figure 2), we found that 60% of **8** was degraded and transformed into two major chemical structures (b and c in Figure 2 and Scheme 2). As shown in the total ion chromatogram in Figure 2B, the mass spectra peaks of the two major degradation products were detected at a mass-to-charge ratio (*m/z*) of 355.2 [M + H]^+^ (Compound b in Figure 2D) and 356.2 [M + H]^+^ (Compound c in Figure 2E) in a positive detection manner. The *m/z* of the two major degradation products was almost identical with that of Compound **8** (a: *m/z* 357.2 [M + H]^+^ in Figure 2C), which indicated that the degraded molecules lost only one or two hydrogen atoms while retaining the complete molecular skeleton. The degradation mechanism was elucidated and is shown in Scheme 2. The p-aminophenol segment in the structure of **8** would be easily oxidized in water solution under the presence of hydrogen ion. Therefore, the introduced active segment should be protected to increase the stabilization of Compound **8**. To achieve this, based on the pro-drug theory, Compound **11** was synthesized through acetylating **8** with acetyl chlorine, which could not only protect the p-aminophenol segment, but could also be hydrolyzed by enzymolysis and transform to the original compound *in vivo*. Thus, the stabilization problem was resolved.

### 2.2. Biological Activity

#### 2.2.1. *In Vitro* Anticancer Activity

Anticancer activities of the key isocorydine derivatives and reference compound cisplatin were screened by using the MTT assay in three common human cancer cell lines: liver (HepG2), lung (A549) and gastric (SGC7901) cancer cells [15]. The 50% inhibitory concentration (IC_50_) values of the isocorydine derivatives and positive control compound exposed for 48 h are listed in Table 1. Among the tested compounds, Compounds **8** and **10** showed relatively low IC_50_ values: 56.18 μM, 7.53 μM and 14.80 μM for Compound **8** and 20.42 μM, 8.59 μM and 14.03 μM for Compound **10** in the three cell lines, respectively. Compared to isocorydine (**1**), the pharmacological activities of **8** and **10** were improved significantly. Therefore, **8** and **10** were selected as potent anticancer agents for further researches *in vivo*. While among the isocorydine derivatives, **2** exhibited relatively weak anticancer activities against the three cell lines (IC_50_: 186.97 μM, 197.73 μM and 212.46 μM). The only difference found in the chemical structures of **2** and **10** was the lack of absolute configuration at C-6a, which should therefore be regarded as the key factor affecting the pharmacological activity of aporphine alkaloids.

**Table 1:** Anticancer activities of the key isocorydine derivatives on three common human cancer cell lines.

Compounds	Mean IC_50_ (μM)		
	HepG2	A549	SGC7901
1	>200.00	>200.00	>200.00
2	186.97	197.73	>200.00
6	78.10	63.70	67.91
7	>200.00	>200.00	>200.00
8	56.18	7.53	14.80
9	>200.00	>200.00	>200.00
10	20.42	8.59	14.03
11	>200.00	>200.00	>200.00
Cisplatin	0.67	1.02	0.88

Compound **6**, which was obtained through further modification of **2**, exhibited better anticancer activities against HepG2, A549 and SGC7901 cells (IC_50_: 78.10 μM, 63.70 μM and 67.91 μM, respectively) than **2**. Compounds **7** and **9**, which were prepared by replacing the hydrogen atom of isocorydine at C-8 with nitro and chloride, respectively, showed no inhibitory effects on the selected cancer cell lines, due to their strong hydrophobicity, which limited their effective concentrations in cells cultured in media. As expected, Compound **11**, which was synthesized based on the pro-drug theory [16] to protect the p-aminophenol fragment, also did not show anticancer activity.

#### 2.2.2. *In Vivo* Anticancer Activity

Given the relatively easy preparation and the p-benzoquinonyl pharmacophore of Compound **2**, the pro-drug design and the good antitumor activity *in vitro* of Compound **8**, Compounds **2** and **11** were selected for evaluation of the tumor growth inhibition in tumor-bearing mice at three different dosages (low, medium and high). The *in vivo* anticancer activity of **10** ultimately could not be evaluated, due to its poor solubility in water and low yield. The inhibitory effect of isocorydione (**2**) on murine sarcoma S_180_-bearing mice is detailed in Table 2. The inhibitory rate and tumor weight of the treatment group were significantly different from those of the blank control group (physiological saline). The tumor inhibitory rate increased with increasing dosage (Figure S1). Although the inhibitory rate of **2** was not better than that of the positive control group treated with cyclophosphamide (CTX), the weights of mice treated with **2** were significantly (*p* < 0.05) higher than those of the CTX group.

**Table 2:** Inhibitory effect of isocorydione (**2**) on S_180_-bearing mice.

Treatment group	Mice number	Dose (mg/kg/d)	Tumor weight (g, Mean ± SD)	Inhibition ratio (%)
Control	11	-	1.2917 ± 0.0935	-
2	11	200	0.6431 ± 0.0197 **	50.21
2	11	100	0.7011 ± 0.0480 *	45.72
2	11	50	0.9661 ± 0.0720	25.21
CTX	11	20	0.4645 ± 0.0152 **	64.04

*vs.* the control group: * *p* < 0.05, ** *p* < 0.01.

**Figure 3:**
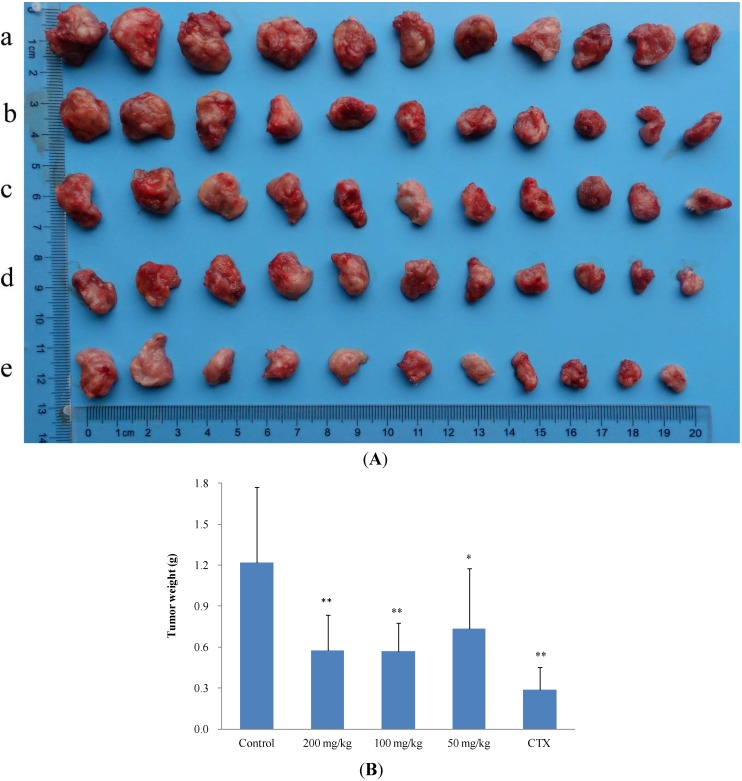
Inhibitory effect of 8-acetamino-isocorydine (**11**) on murine hepatoma H_22_-bearing mice. (**A**) Photograph of the tumors excised from H22-bearing mice (a, blank control group; b, 50 mg/kg group; c, 100 mg/kg group; d, 200 mg/kg group; e, CTX group). From the perspective of gross anatomy, the tumor size of treatment groups and the CTX group were all significantly different from that of the control group; (**B**) The mean tumor weights (X + SD) of all groups. The tumor weights of the high dosage group, the medium dosage group and the CTX group were all significantly different from that of the blank control group (** *p* < 0.01, * *p* < 0.05).

**Figure 4:**
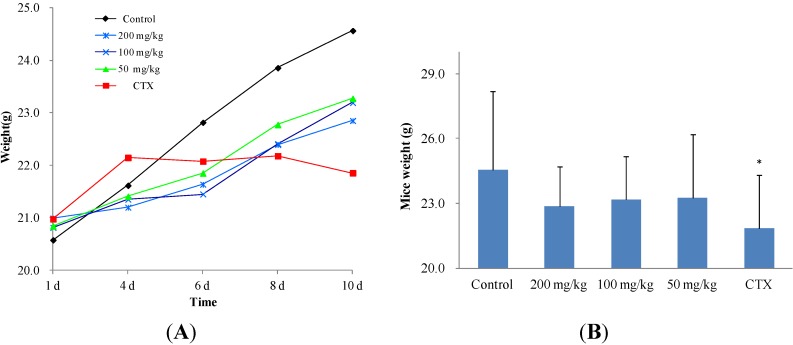
Influence of 8-acetamino-isocorydine (**11**) on body weight of mice bearing murine hepatoma H_22_. (**A**) The trend of weight increase during the treatment period with **11**. The body weights increased in the blank control and treatment groups, but decreased in the CTX group; (**B**) Body weight of all groups at 10 d. The body weight of the blank control group and treatment groups were significantly different from that of the CTX group (* *p* < 0.05). There was no significant difference between the blank control group and treatment groups.

In preliminary experiments, the tumor growth inhibitory effect of **11** on S_180_-bearing mice was relatively weak (Figure S2). Therefore, the murine hepatoma H_22_ mouse model was selected for this investigation. The inhibitory effect of **11** on H_22_ tumors is detailed in Table 3. The inhibitory rate of the high-dose group and the medium-dose group were similar (52.71% and 53.12%, respectively); however, the tumor weight and size of the CTX group and the three dosage groups were significantly different from those of the control group (Figure 3A,B). Compared to CTX, the inhibitory effect of Compound **11** was not strong, but the mean body weight of the CTX group was significantly (*p* < 0.05) lower than those of the dosage groups and control group. In particular, there was a declining trend of mouse body weight in the CTX group during the 10-d treatment period (Figure 4A,B), which would be a dangerous signal in clinical chemotherapy. Thus, it was concluded that **2** and **11** have significant antitumor activity by reducing both tumor size and weight *in vivo*. It is worth noting that no difference in mouse body weight was observed between the treatment groups and the blank control group, suggesting that the isocorydine derivatives had no adverse side effects on mouse growth as observed in the CTX group. Therefore, 8-acetamino-isocorydine (**11**) should be further investigated as a potential antitumor agent.

**Table 3:** Inhibitory effect of 8-acetamino-isocorydine (**11**) on H_22_-bearing mice.

Treatment group	Mice number	Dose (mg/kg/d)	Tumor weight (g, Mean ± SD)	Inhibition ratio (%)
Control	11	-	1.2189 ± 0.5520	-
11	11	200	0.5764 ± 0.2615 **	52.71
11	11	100	0.5714 ± 0.2087 **	53.12
11	11	50	0.7359 ± 0.4401 *	39.63
CTX	11	20	0.2902 ± 0.1610 **	76.19

*vs*. the control group: * *p* < 0.05, ** *p* < 0.01.

### 2.3. The Structure and Activity Relationships of ICD Alkaloid

In this research, the structure activity shows that different substituents at C-8 position can significantly affect the anticancer activities on test cell lines. That is, compounds that have strong electro donating group (-NH_2_) at the C-8 position have better *in vitro* anticancer activity than that of those that have electro withdrawing groups (-NO_2_) and weak electro donating groups (-Cl) at the C-8 position.

The anticancer activity of **2** was superior to that of isocorydine (**1**), due to the p-benzoquinone fragment in its D ring, which could expand the conjugated system. As a biphenyl, isocorydine has a nearly coplanar and hyperconjugated rigid chemical skeleton, and its conjugated structure would be favorable to enhance the planarity of a targeted molecule, which could easily be intercalated into the DNA double helix and block DNA replication, thereby inhibiting cancer cell growth, which has been confirmed in the literature (Figure 1) [17,18]. However, although the chemical structure planarity of **2** was superior to that of **10**, **2** showed weaker anticancer activity compared to **10** *in vitro*. This was likely due to the dehydrogenation reaction between C-6a and C-7 in **2** and not **10**, which resulted in loss of the hydrogen atom at C-6a that affected the absolute configuration. Thus, the absolute configuration of the hydrogen atom at C-6a appears to be a key factor in the anticancer activity of these aporphine alkaloids, because the orientation of the hydrogen atom at C-6a determines the orientation of the lone pair of electrons of the nitrogen atom.

## 3. Experimental

### 3.1. Chemistry

#### 3.1.1. Materials

Melting points were obtained on an X-4 digital display micro-melting point apparatus and uncorrected. NMR spectra were recorded on a Varian INOVA-400 MHz-FTNMR spectrometer with TMS as the internal standard. HRMS (ESI) was recorded on a Bruker APEX II. The HPLC system was an Agilent 1100 series with an autosampler and column heater (Palo Alto, CA, USA). The MS system was an Agilent LC-MSD-Trap (Palo Alto, CA, USA). An accurate post-column splitter from LC Packing (San Francisco, CA, USA) was used between the outlet of the UV detector and the inlet of the MS detector. Agilent MSD Trap control software version 5.0 (Palo Alto, CA, USA) was used for MS data acquisition and data processing. Chromatographic conditions for HPLC-UV-MS methods: UV detector settled at 270 nm, a SinoCrom ODS-BP C18 column (4.6 mm × 250 mm, 5 μm) was used with the mobile phase consisting of MeOH:water (65:35, using aqua ammonia adjusted pH at 7.2) at a flow rate of 1.0 mL/min. The column temperature was maintained at 25 °C. The sample solution was injected at a concentration of approximately 0.3 mg/mL with an injection volume of 20 μL. MS detection using ESI, operated in positive mode, was used to help identify molecular ions and track unknown degradation. The trap drive was set at 69 V, drying temperature at 325 °C, nebulizer gas at 8.0 psi, drying gas flow at 5.00 L/min, scan speed at *m/z* 13,000 s^−1^ and scan range (*m/z*) at 50–2200.

Column chromatography was performed on silica gel (200–300 mesh, Qingdao Puke Parting Materials Co., Ltd., Qingdao, China). TLC was carried out on silica gel GF254 (Qingdao Marine Chemical Ltd., Qingdao, China), and spots were visualized under UV light or by spraying Dragendorff’s reagent (Beijing, China). The Pd/C (10%) catalyst was purchased from Sinopharm Chemical Reagent Co., Ltd (Shanghai, China).

The whole plants of *D. leptopodum* (Maxim) Fedde were collected in Pingliang of Gansu Province, China and identified by Prof. Zhigang Ma (Lanzhou University). A voucher specimen had been deposited at Key Laboratory of Chemistry of Northwestern Plant Resources and Key Laboratory for Natural Medicine of Gansu Province, Lanzhou Institute of Chemical Physics, Chinese Academy of Sciences, Lanzhou, China.

### 3.2. General Procedure for ICD Derivatives Preparations

#### 3.2.1. Isolation of ICD (**1**)

Whole plants of *D. leptopodum* (Maxim) Fedde (200 kg) were smashed and extracted twice with 95% ethanol (1000 L) at 60 °C for 2.5 h. The mixture was filtered. The filtrate was combined and evaporated by a rotary evaporator. The residue (24.0 kg) was dissolved in 2% H_2_SO_4_ aqueous solution (200 L). The solution was extracted with chloroform (200 L × 3). The chloroform parts were concentrated by a rotary evaporator and dried by a vacuum oven at 30 °C to give Extract A (5.0 kg). The pH of the aqueous solution layer was adjusted with NH_4_OH to **9**, and this solution was also extracted with chloroform to give Extract B (2.0 kg). Extract B was separated by silica gel column (200 cm × 2 m) and was gradient eluted with chloroform/MeOH/(Et)_2_NH (10:1:0.1 to 2:1:0.1). Four fractions, B_1_ (400 g, 100:1), B_2_ (900 g, 50:1), B_3_ (500 g, 25:1) and B_4_ (200 g, 5:1), were obtained. Then, B_2_ was further separated by silica gel column to give ICD (**1**) 250.0 g, which has been recrystallized from methanol to obtain colorless prism crystal. M.p.: 185–186 °C; 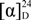 + 205.0 (c 0.1, MeOH); ^1^H-NMR (400 MHz, CD_3_COCD_3_) δ: 6.74 (1H, s, H-3), 3.36 (2H, m, H-4), 2.49 (2H, m, H-5), 2.61 (1H, dd, *J* = 16.4, 3.6 Hz, H-6a), 2.99 (2H, m, H-7), 7.00 (1H, d, *J* = 8.0 Hz, H-8), 7.14 (1H, d, *J* = 8.0 Hz, H-9), 3.04 (3H, s, N-CH_3_), 3.67 (3H, s, 1-OCH_3_), 3.88 (3H, s, 2-OCH_3_), 3.79 (3H, s, 10-OCH_3_); ^13^C-NMR (100 MHz, CD_3_COCD_3_) δ: 142.5 (C-1), 124.2 (C-1a), 126.2 (C-1b), 152.0 (C-2), 111.4 (C-3), 130.7 (C-3a), 35.9 (C-4), 52.7 (C-5), 63.0 (C-6a), 29.5 (C-7), 128.6 (C-7a), 119.4 (C-8), 112.1 (C-9), 149.1 (C-10), 144.1 (C-11), 124.3 (C-11a), 65.5 (1-OCH_3_), 55.4 (2-OCH_3_), 52.7 (10-OCH_3_), 43.4 (N-CH_3_). HRMS (ESI): *m/z* calcd. for C_20_H_24_NO_4_ [M + H]^+^ 342.1699, obsd. 342.1647.

#### 3.2.2. Synthesis of Isocorydione (**2**)

Compound **2** was synthesized from isocorydine by using a method described previously [15]. Chopped ice (200.0 g) was added to a solution of sodium nitrite (5 M, 100 mL) at 0 °C. The solution was stirred steadily while adding fresh sodium bisulfate solution (100 mL, 35% w/v), followed by glacial acetic acid (20 mL). After stirring for 2–3 min, the color of the reaction mixture became dark, and concentrated ammonia solution (25 mL) was added. Subsequently, ice-cold potassium permanganate solution (0.2 M, 400 mL) was added in a drop-wise manner over 1 h. When the color of the reaction mixture became dark brown, the reaction was terminated. Manganese dioxide, which was generated in the reaction, was filtered rapidly. A portion of the filtrate (10–15 mL) was treated with an equal volume of saturated potassium chloride solution to prepare Fremy salt as a seed crystal. Saturated potassium chloride solution (250 mL) was added in a drop-wise manner to the remaining filtrate over a period of approximately 45 min. Subsequently, the previously prepared Fremy salt was added to the solution periodically until a solid appeared in the bulk solution. Then, the bulk solution was stirred in an ice bath for a further 45 min. The orange solid (Fremy salt) was collected under vacuum filtration, and the solid was washed with an ammoniacal saturated potassium chloride solution (containing ~5% v/v 0.88 ammonium hydroxide), then with ammoniacal methanol (containing ~5% v/v 0.88 ammonium hydroxide) and, finally, with acetone.

To a solution of disodium hydrogen phosphate (2.5 g) in water (750 mL), Fremy radical (12.0 g) was added followed by **1** (5.0 g). The reaction mixture was stirred overnight at room temperature. Then, the reaction solution was extracted with chloroform (200 mL × 3). The chloroform layers were combined, dried with Na_2_SO_4_ and evaporated by rotary evaporation under reduced pressure. The residue was purified through column chromatography on a silica gel (petroleum ether (60–90 °C): acetone, 3:1) to give isocorydione (**2**) (2.4 g, yield of 50%). Compounds **3**, **4** and **5** were by-products obtained during the synthesis of **2** and were also isolated from the chloroform residue through column chromatography, with yields of 1.0%, 2.0% and 5.0%, respectively. Compound **2** is a purple amorphous powder (carbinol); purity: 98.0%; solubility: soluble in organic solvent and insoluble in water; m.p.: 198–200 °C; ^1^H-NMR (400 MHz, CDCl_3_): 6.93 (1H, s, H-3), 3.13 (2H, t, *J* = 6.4 Hz, H-4), 3.47 (2H, t, *J* = 6.4 Hz, H-5), 6.95 (1H, s, H-7), 5.90 (1H, s, H-9), 3.17 (3H, s, N-CH_3_), 3.97 (3H, s, 1-OCH_3_), 3.87 (3H, s, 2-OCH_3_), 3.94 (3H, s, 10-OCH_3_); ^13^C-NMR (100 MHz, CDCl_3_): 143.8 (C-1), 126.9 (C-1a), 119.3 (C-1b), 152.2 (C-2), 112.8 (C-3), 128.3 (C-3a), 29.2 (C-4), 50.2 (C-5), 150.3 (C-6a), 98.3 (C-7), 136.5 (C-7a), 186.5 (C-8), 105.1 (C-9), 163.8 (C-10), 178.3 (C-11), 117.9 (C-11a), 60.7 (1-OCH_3_), 56.5 (2-OCH_3_), 56.4 (10-OCH_3_), 40.2 (N-CH_3_). HRMS (ESI): *m/z* calcd. for C_20_H_20_NO_5_ [M + H]^+^ 354.1336, obsd. 354.1335.

#### 3.2.3. 4-Hydroxy-isocorydione (**3**)

Purple amorphous powder (carbinol); purity: 95.0%; solubility: soluble in organic solvent and insoluble in water; ^1^H-NMR (400 MHz, CDCl_3_): 6.93 (1H, s, H-3), 4.92 (2H, t, *J* = 6.4, 2.1 Hz, H-4), 3.62 (2H, t, *J* = 6.4 Hz, H-5), 7.18 (1H, s, H-7), 5.84 (1H, s, H-9), 3.19 (3H, s, N-CH_3_), 3.94 (3H, s, 1-OCH_3_), 3.87 (3H, s, 2-OCH_3_), 3.85 (3H, s, 10-OCH_3_); ^13^C-NMR (100 MHz, CDCl_3_): 145.0 (C-1), 126.6 (C-1a), 119.1 (C-1b), 152.2 (C-2), 112.7 (C-3), 129.9 (C-3a), 66.6 (C-4), 57.1 (C-5), 149.1 (C-6a), 98.8 (C-7), 136.2 (C-7a), 186.1 (C-8), 105.2 (C-9), 163.8 (C-10), 178.5 (C-11), 118.0 (C-11a), 60.7 (1-OCH_3_), 56.3 (2-OCH_3_), 56.1 (10-OCH_3_), 40.2 (N-CH_3_). HRMS (ESI): *m/z* calcd. for C_20_H_20_NO_6_ [M + H]^+^ 370.1285, obsd. 370.1289.

#### 3.2.4. N-demethyl-isocorydione (**4**)

Purple amorphous powder (carbinol); purity: 95.0%; solubility: soluble in organic solvent and insoluble in water; ^1^H-NMR (400 MHz, CDCl_3_): 6.95 (1H, s, H-3), 3.13 (2H, t, *J* = 6.4 Hz, H-4), 3.55 (2H, t, *J* = 6.4 Hz, H-5), 6.98 (1H, s, H-7), 5.89 (1H, s, H-9), 3.98 (3H, s, 1-OCH_3_), 3.88 (3H, s, 2-OCH_3_), 3.96 (3H, s, 10-OCH_3_); ^13^C-NMR (100 MHz, CDCl_3_): 143.9 (C-1), 126.7 (C-1a), 118.5 (C-1b), 152.6 (C-2), 113.3 (C-3), 128.7 (C-3a), 29.2 (C-4), 40.7 (C-5), 149.5 (C-6a), 100.7 (C-7), 136.5 (C-7a), 186.1 (C-8), 105.2 (C-9), 164.0 (C-10), 178.4 (C-11), 118.0 (C-11a), 60.7 (1-OCH_3_), 56.5 (2-OCH_3_), 56.4 (10-OCH_3_). HRMS (ESI): *m/z* calcd. for C_19_H_18_NO_5_ [M + H]^+^ 340.1179, obsd. 340.1183.

#### 3.2.5. 7-Hydroxy-N-demethyl-isocorydione (**5**)

Red amorphous powder, purity: 95.0%; solubility: soluble in organic solvent and insoluble in water; ^1^H-NMR (400 MHz, CDCl_3_): 7.21 (1H, s, H-3), 7.73 (1H, d, *J* = 5.2 Hz, H-4), 8.83 (1H, d, *J* = 5.2 Hz, H-5), 6.67 (1H, s, H-9), 4.07 (3H, s, 1-OCH_3_), 3.84 (3H, s, 2-OCH_3_), 4.01 (3H, s, 10-OCH_3_); ^13^C-NMR (100 MHz, CDCl_3_): 146.2 (C-1), 117.3 (C-1a), 121.8 (C-1b), 155.8 (C-2), 105.9 (C-3), 135.6 (C-3a), 122.7 (C-4), 145.4 (C-5), 145.3 (C-6a), 163.3 (C-7), 109.4(C-7a), 184.2 (C-8), 101.5 (C-9), 159.1 (C-10), 138.8 (C-11),120.6 (C-11a), 63.2 (1-OCH_3_), 56.6 (2-OCH_3_), 56.4 (10-OCH_3_). HRMS (ESI): *m/z* calcd. for C_19_H_17_NO_6_ [M + H]^+^ 354.0972, obsd. 354.0976.

#### 3.2.6. 8-Hydroxylamine-isocorydione (**6**)

To a solution of **2** (1.0 g) in absolute ethyl alcohol (150 mL), sodium hydroxide (1.3 g) was added followed by hydroxyl ammonium chloride (3.2 g). The reaction mixture was refluxed at 80 °C for 1.5 h. Then, the solvent was evaporated by rotary evaporation under reduced pressure. The crude product was purified by column chromatography on a silica gel (chloroform: methanol, 10:1) to give 8-hydroxylamine-isocorydione (**6**) with a yield of 90%. Compound **6** is a brown crystal, purity: 99.0%; solubility: soluble in organic solvent and slightly soluble in water; ^1^H-NMR (400 MHz, DMSO-*d_6_*): 6.80 (1H, s, H-3), 3.14 (2H, t, H-4), 3.47 (2H, t, H-5), 7.18 (1H, s, H-9), 3.16 (3H, s, N-CH_3_), 3.90 (3H, s, 1-OCH_3_), 3.73 (3H, s, 2-OCH_3_), 3.91 (3H, s, 10-OCH_3_), 11.90 (1H, s, -OH); ^13^C-NMR (100 MHz, DMSO-*d_6_*): 142.7 (C-1), 126.5 (C-1a), 119.3 (C-1b), 151.3 (C-2), 112.1 (C-3), 128.7 (C-3a), 28.6 (C-4), 49.5 (C-5), 139.1 (C-6a), 147.8 (C-7a), 148.9 (C-8), 94.1 (C-9), 150.3 (C-10), 179.8 (C-11), 118.1 (C-11a), 59.4 (1-OCH_3_), 56.2 (2-OCH_3_), 40.1 (10-OCH_3_), 39.9 (N-CH_3_). HRMS (ESI): *m/z* calcd. for C_20_H_22_N_2_O_5_ [M + 2H]^+^ 370.1397, obsd. 370.1398.

#### 3.2.7. Synthesis of 8-Nitro-isocorydine (**7**)

A solution of isocorydine (3.0 g) in dichloromethane (200 mL) was treated with a nitration reagent, which was composed of fuming nitric acid (0.74 mL) and concentrated sulfuric acid (98%, 0.74 mL) at −30 °C. After stirring for 40 min, the reaction mixture was poured into ice water and alkalified to pH 8 with ammonia water, and the solution was extracted three times with chloroform. The chloroform part was concentrated by rotary evaporation under reduced pressure. The residue was purified by column chromatography on a silica gel (chloroform: methanol, 20:1) to give 8-nitro-isocorydine (**7**), with a yield of 51%. Compound **7** is a yellow needle crystal, purity: 99.0%; solubility: soluble in organic solvent and insoluble in water; 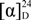 + 170.0 (c 0.1, MeOH); ^1^H-NMR (400 MHz, CDCl_3_): 6.76 (1H, s, H-3), 3.00 (1H, m, H-4), 2.40–2.48 (1H, *J* = 17.2, 2.8 Hz, H-4), 2.68–2.72 (2H, dd, *J* = 17.2, 2.8 Hz, H-5), 3.67 (1H, dd, *J* = 14.4, 3.2 Hz, H-6a), 3.00–3.21 (2H, m, H-7), 7.52 (1H, s, H-9), 3.46 (3H, s, N-CH_3_), 3.71 (3H, s, 1-OCH_3_), 3.96 (3H, s, 2-OCH_3_), 3.90 (3H, s, 10-OCH_3_); ^13^C-NMR (100 MHz, CDCl_3_): 142.3 (C-1), 128.0 (C-1a), 130.2 (C-1b), 151.4 (C-2), 112.1 (C-3), 127.5 (C-3a), 29.1 (C-4), 52.6 (C-5), 62.4 (C-6a), 31.0 (C-7), 121.3 (C-7a), 141.8 (C-8), 107.1 (C-9), 148.8 (C-10), 148.7 (C-11), 123.8 (C-11a), 61.6 (1-OCH_3_), 56.3 (2-OCH_3_), 55.9 (10-OCH_3_), 43.9 (N-CH_3_). HRMS (ESI): *m/z* calcd. for C_20_H_23_N_2_O_6_ [M + H]^+^ 387.1551, obsd. 387.1565.

#### 3.2.8. 8-Amino-isocorydine (**8**)

The mixture of Compound **7** (1.0 g) with palladium on carbon hydrogenation catalyst (10%, 0.5 g) in absolute ethyl alcohol (400 mL) was stirred for 1.5 h, with hydrogen pressure maintained at 0.3 MPa in a high-pressure autoclave. Then, the mixture was filtered to remove the catalyst. The filtrate was evaporated by rotary evaporation under reduced pressure. The residue was purified by column chromatography on a silica gel (chloroform:methanol, 5:1) to give 8-*amino*-isocorydine (**8**), with a yield of 80%, which is a brown amorphous powder; purity: 98.0%; solubility: soluble in organic solvent and in water; 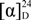 + 170.0 (c 0.1, MeOH);^1^H-NMR (400 MHz, CDCl_3_): 6.60 (1H, s, H-3), 3.00 (2H, m, H-4), 2.43 (2H, m, H-5), 3.00 (1H, m, H-6a), 2.88 (1H, dd, *J* = 16.4, 2.0 Hz, H-7), 2.60 (1H, dd, *J* = 16.4, 2.0 Hz, H-7), 4.35 (2H, s, 8-NH_2_), 6.34 (1H, s, H-9), 2.51 (3H, s, N-CH_3_), 3.56 (3H, s, 1-OCH_3_), 3.79 (3H, s, 2-OCH_3_), 3.76 (3H, s, 10-OCH_3_); ^13^C-NMR (100 MHz, CDCl_3_): 142.2 (C-1), 125.9 (C-1a), 128.9 (C-1b), 151.2 (C-2), 113.0 (C-3), 127.6 (C-3a), 29.2 (C-4), 52.2 (C-5), 62.2 (C-6a), 36.6 (C-7), 120.6 (C-7a), 135.4 (C-8), 102.0 (C-9), 149.2 (C-10), 136.9 (C-11), 110.7 (C-11a), 62.8 (1-OCH_3_), 55.8 (2-OCH_3_), 55.4 (10-OCH_3_), 43.9 (N-CH_3_). HRMS (ESI): *m/z* calcd. for C_20_H_25_N_2_O_4_ [M + H]^+^ 357.1809, obsd. 357.1816.

#### 3.2.9. 8-Chloro-isocorydine (**9**)

N-chlorosuccinimide (1.2 g) was added to a stirred solution of isocorydine (3.0 g) in glacial acetic acid (150 mL). After vigorous stirring for 2 h at 7 °C, the reaction mixture was poured into ice, alkalified to pH 8 with ammonia water and extracted three times with chloroform. The organic part was combined and evaporated by rotary evaporation under reduced pressure. The residue was purified by column chromatography on a silica gel (chloroform: methanol, 20:1) to give 8-chloro-isocorydine (**9**), with a yield of 60%, which is a yellow amorphous powder. Purity: 98.0%; solubility: soluble in organic solvent and insoluble in water; 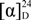 + 175.0 (c 0.1, MeOH) ^1^H-NMR (400 MHz, CDCl_3_): 6.74 (1H, s, H-3), 2.31–2.31 (2H, m, H-4), 2.91–2.95 (2H, m, H-5), 3.06 (1H, m, H-6a), 2.67–2.69 (2H, m, H-7), 7.50 (1H, s, H-9), 2.38 (3H, s, N-CH_3_), 3.60 (3H, s, 1-OCH_3_), 3.85 (3H, s, 2-OCH_3_), 3.79 (3H, s, 10-OCH_3_); ^13^C-NMR (100 MHz, CDCl_3_): 142.1 (C-1), 128.5 (C-1a), 130.1 (C-1b), 151.2 (C-2), 112.0 (C-3), 127.4 (C-3a), 29.6 (C-4), 52.5 (C-5), 62.3 (C-6a), 30.9 (C-7), 121.2 (C-7a), 141.7 (C-8), 107.0 (C-9), 148.3 (C-10), 148.7 (C-11),123.7 (C-11a), 61.5 (1-OCH_3_), 56.3 (2-OCH_3_), 55.9 (10-OCH_3_), 43.9 (N-CH_3_). HRMS (ESI): *m/z* calcd. for C_20_H_22_NO_4_Cl [M]^+^ 375.1237, obsd. 375.2060.

#### 3.2.10. 6a,7-Dihydrogen-isocorydione (**10**)

Sodium nitrite (1.3 g) was added into the water (250 mL) solution of 8 (3.4 g) and then hydrochloric acid (1.2 mL) was dropped in the reaction bulb. The mixture was stirred for 1 h at 0 °C. Then, the reaction was quenched by adding a solution of urea (10%). The reaction solution was extracted with 1,2-dichloroethane (200 mL × 3). The organic layers were combined and evaporated by rotary evaporation. The crude product was then purified by column chromatography on a silica gel (chloroform: methanol, 20:1) to give 6a, 7-dihydrogen-isocorydione (**10**), with a yield of 20%, which is a brown amorphous powder. Purity: 99.0%; solubility: soluble in organic solvent and insoluble in water; ^1^H-NMR (400 MHz, CDCl_3_): 6.67 (1H, s, H-3), 5.88 (1H, s, H-9), 3.84 (3H, s, 1-OCH_3_), 3.82 (3H, s, 10-OCH_3_), 3.79 (3H, s, 2-OCH_3_), 2.46 (3H, s, N-CH_3_), 3.34 (1H, m, H-6a), 3.00 (2H, m, H-5), 2.42 (1H, m, H-4), 2.85 (1H, dd, *J* = 16.4 Hz, H-7), 2.60 (1H, dd, *J* = 16.4 Hz, H-7); ^13^C-NMR (100 MHz, CDCl_3_): 140.8 (C-1), 127.9 (C-1a), 121.4 (C-1b), 151.8 (C-2), 114.3 (C-3), 128.4 (C-3a), 28.4 (C-4), 53.3 (C-5), 61.3 (C-6a), 25.6 (C-7), 185.5 (C-8), 106.1 (C-9), 160.1 (C-10), 180.2 (C-11), 127.8 (C-11a), 60.9 (1-OCH_3_), 56.3 (2-OCH_3_), 55.7 (10-OCH_3_), 43.9 (N-CH_3_). HRMS (ESI): *m/z* calcd. for C_20_H_21_NO_5 _ [M + H]^+^ 357.1576, obsd. 357.1534.

#### 3.2.11. 8-Acetamino-isocorydine (**11**)

Ten milliliters fresh distilled acetyl chloride were added to a mixture of **8** (1.0 g) and 10 mL acetic ether in a round-bottomed flask, under vigorous stirring, and then the mixture was stirred at room temperature for 2 h. Then, the reaction mixture was concentrated *in vacuo* to obtain a purple solid. The solid was then purified by column chromatography on a silica gel (chloroform: methanol, 5:1) to give 8-acetamino-isocorydine (**11**), with a yield of 80%, which is a brown amorphous powder. Purity: 99.0%; solubility: soluble in organic solvent and in water; 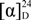 + 172.0 (c 0.1, MeOH); ^1^H-NMR (400 MHz, CD_3_Cl): 7.53 (1H, s, H-3), 3.00 (2H, m, H-4), 2.43 (2H, m, H-5), 3.00 (1H, m, H-6_a_), 2.88 (1H, dd, *J* = 16.4, 2.0 Hz, H-7), 2.60 (1H, dd, *J* = 16.4, 2.0 Hz, H-7), 6.73 (1H, s, H-9), 2.48 (3H, s, N-CH_3_), 3.43 (3H, s, 1-OCH_3_), 3.90 (3H, s, 2-OCH_3_), 3.86 (3H, s, 10-OCH_3_); 2.22 (3H, s, COCH_3_). ^13^C-NMR (100 MHz, CD_3_Cl): 146.3 (C-1), 128.0 (C-1a), 128.5 (C-1b), 151.9 (C-2), 113.2 (C-3), 126.4 (C-3a), 29.4 (C-4), 52.7 (C-5), 61.8 (C-6a), 31.0 (C-7), 122.3 (C-7a), 141.4 (C-8), 107.1 (C-9), 150.9 (C-10), 146.1 (C-11),123.8 (C-11a), 61.3 (1-OCH_3_), 56.1 (2-OCH_3_), 55.5 (10-OCH_3_), 43.6 (N-CH_3_), 21.1 (COCH_3_). HRMS (ESI): *m/z* calcd. for C_22_H_27_N_2_O_5_ [M + H]^+^ 399.1914, obsd. 399.1911.

### 3.3. Biological Assay

#### 3.3.1. Materials

The human lung cancer cells (A549), human gastric cancer cells (SGC7901) and human liver cancer cells (HepG2) were provided by the Cell Bank of the Institute of Biochemistry and Cell Biology at the China Academy of Sciences (Shanghai, China). Murine sarcoma cell line S_180_ and murine hepatoma cell line H_22_ were obtained from cell library of Institute of Cancer Biology and Drug Discovery, Lanzhou University. All cell lines were cultured in RPMI 1640 medium supplemented with 10% heat-inactivated fetal bovine serum (FBS), streptomycin (100 μg/mL) and penicillin (100 U/mL) in a humidified atmosphere containing 5% CO_2_ at 37 °C. Kunming male mice weighing 18.0–22.0 g were purchased from the Experimental Animal Center at Lanzhou University. The use and treatment of mice were in accordance with institutional guideline for Laboratory Animal Care.

#### 3.3.2. Anticancer Activity *in Vitro*

Cell proliferation was analyzed by a colorimetric MTT assay. Cells were seeded in 96-well plates at a density of 5 × 10^3^ cells/well in 100 μL RPMI 1640 medium supplemented with 10% FBS. After 12 h, cells were treated with different concentrations of selected compounds in complete medium, respectively, while the negative control was treated with complete RPMI 1640 medium only. After 48 h, 20 μL MTT (5 mg/mL) was added. After the plates were incubated at 37 °C for 4 h, the supernatant was removed, and then, 150 μL DMSO were added to each well. Absorbance was measured at 570 nm by a 96-well microplate reader (DNM-9602).

#### 3.3.3. Anticancer Activity *in Vivo*

Zero-point-two milliliters of model mice ascites, including 2 × 10^7^ S_180_ or H_22_ cells/mL, were injected into the right axilla of mice. From the second day after the implantation of cancer cells, 55 tumor-bearing mice were grouped randomly into five groups as the following: the blank control group was treated by physiological saline (0.9%), and the positive control group was treated with cyclophosphamide (CTX) at a dosage of 20 mg/kg. Medicated groups were intragastrically administered with targeted compound at 50 mg/kg, 100 mg/kg and 200 mg/kg, respectively. Subsequently, mice were treated daily for the following 10 days. Twenty four hours after the last drug administration, mice were executed, and their tumors were totally excised and accurately weighed. The anticancer activity of ICD derivatives *in vivo* were expressed as an inhibitory rate, which was calculated with the formula: ((mean tumor weight of control group − mean tumor weight of dose group)/mean tumor weight of control group) × 100%.

## 4. Conclusions

Ten ICD derivatives have been prepared through chemical structure modification, and their anticancer activities of the key derivatives have been investigated through *in vitro* and *in vivo* experiments. The results suggested that structural modifications could significantly improve the anticancer activity of ICD alkaloid and have lower side effects on body weight than that of CTX, which indicated that ICD can be a lead compound for the development of an effective anticancer agent.

## Supplementary Materials

Supplementary materials can be accessed at: http://www.mdpi.com/1420-3049/19/8/12099/s1.

## Supplementary Files

Supplementary File 1
