# Supplementary material for: Isocorydine Derivatives and Their Anticancer Activities
Source: Molecules. 2014 Aug 12;19(8):12099–115. doi: 10.3390/molecules190812099 (PMC6271052; doi:10.3390/molecules190812099)

Supplementary

Figure S1. Inhibitory effect of isocorydione (**2**) on murine sarcoma S_{180}-bearing mice (**A**, blank control group; **B**, 50 mg/kg group; **C**, 100 mg/kg group; **D**, 200 mg/kg group; **E**, CTX group).

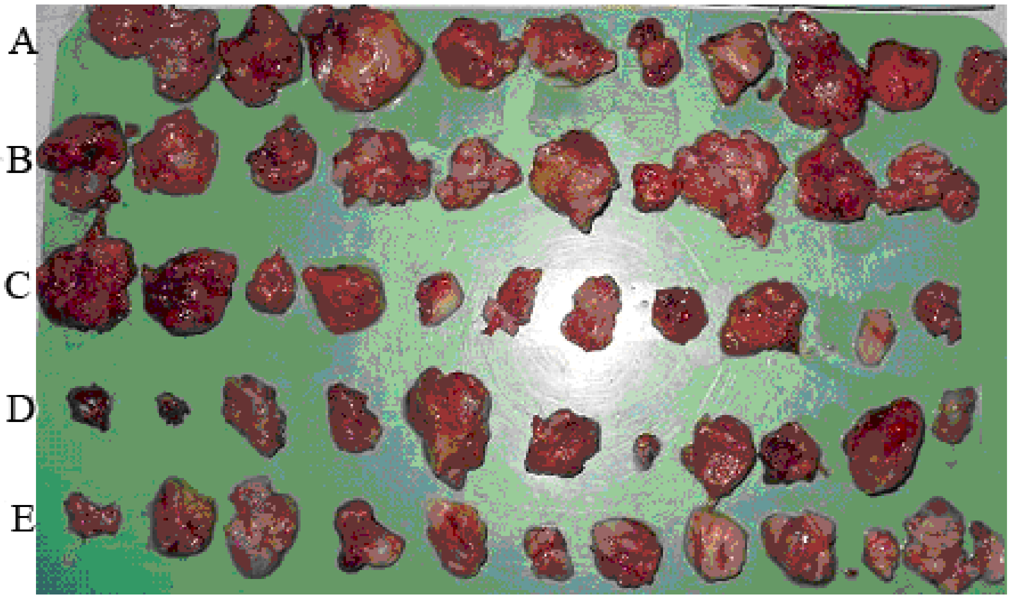

Figure S2. Inhibitory effect of 8-acetamino-isocorydine (**11**) on murine sarcoma S_{180}-bearing mice (**A**, blank control group; **B**, CTX group; **C**, 50 mg/kg group; **D**, 100 mg/kg group; **E**, 200 mg/kg group).

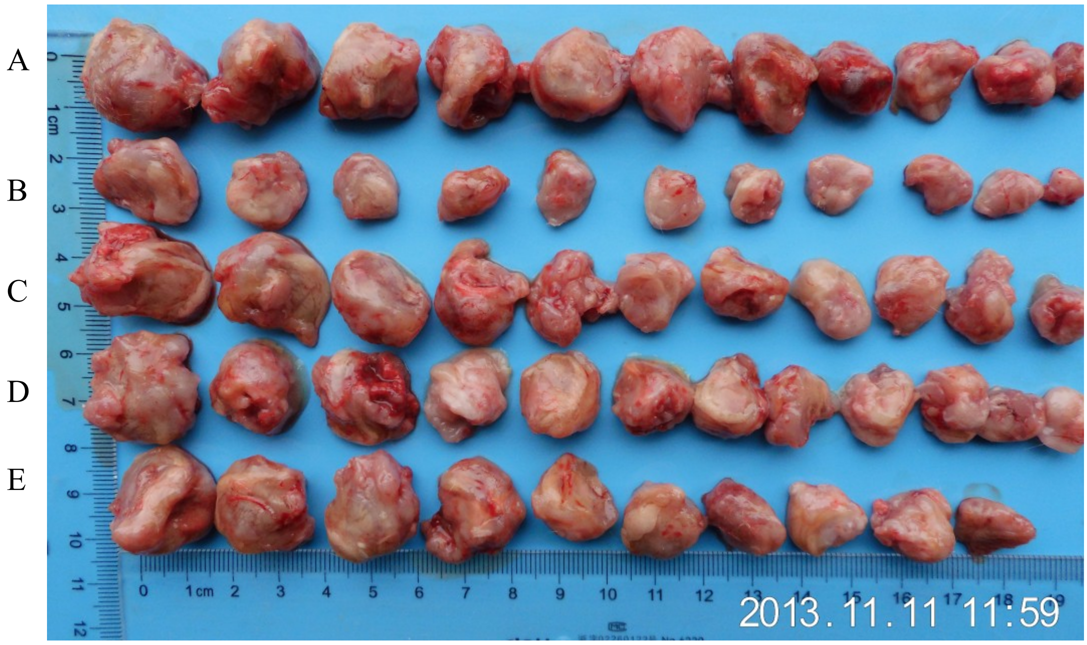

Figure S3. ^{1}H-NMR (400 MHz, CD_3COCD_3) spectra of isocorydine (**1**).

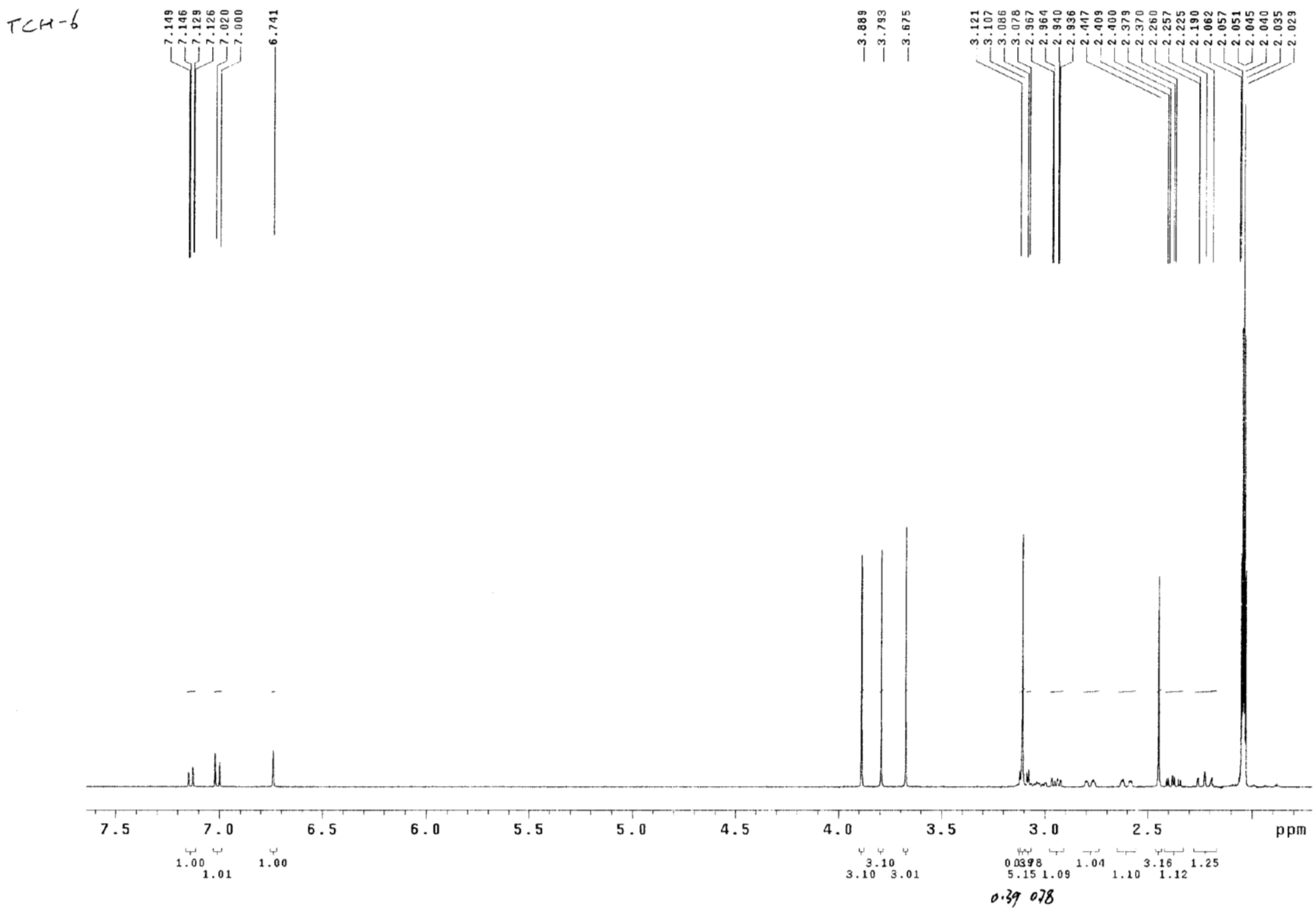

Figure S4. ^{13}C-NMR (100 MHz, CD_3COCD_3) spectra of isocorydine (**1**).

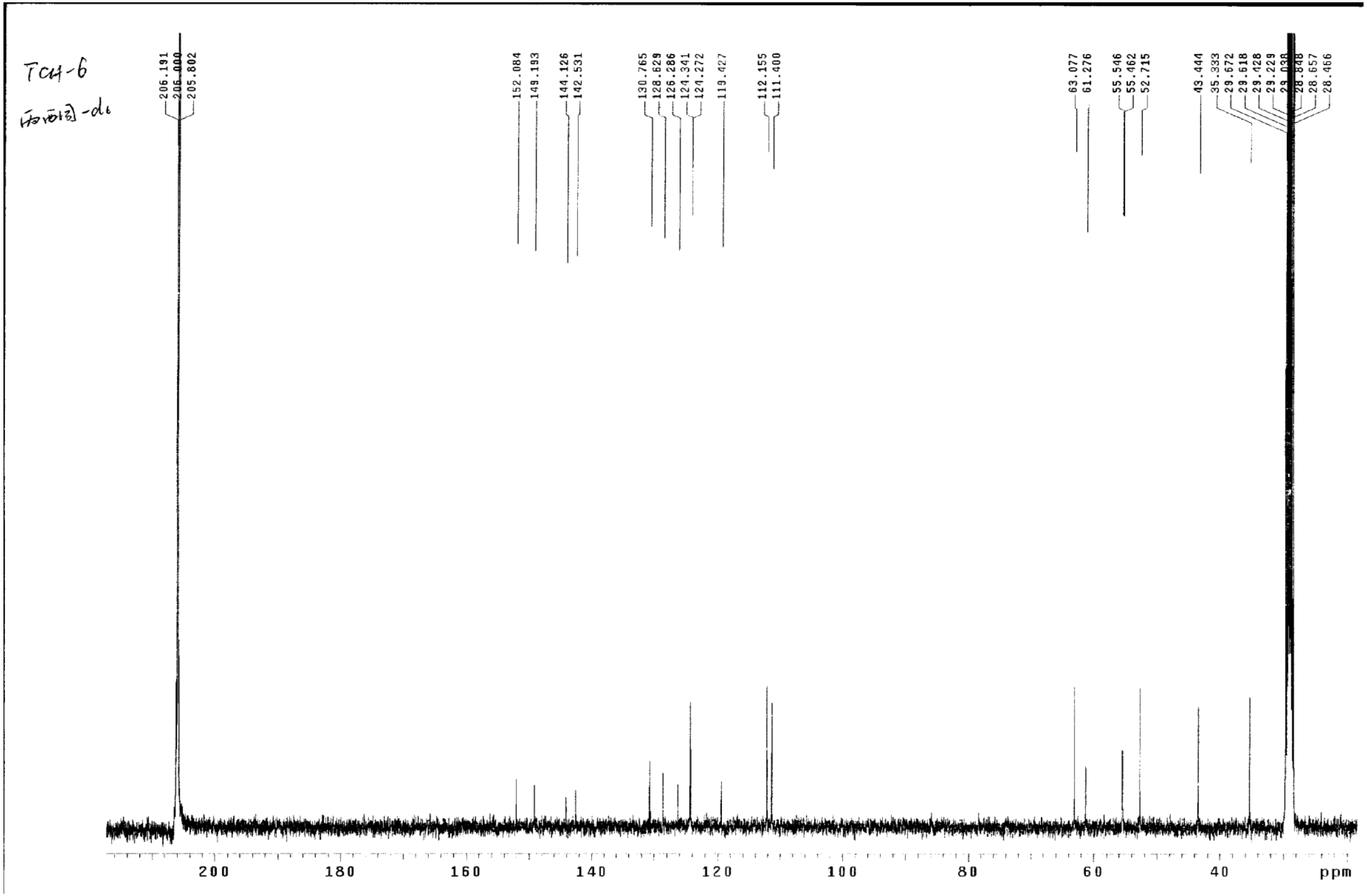

Figure S5. HRMS (ESI) spectra of isocorydine (**1**).

Generic Display Report

Analysis Info

Analysis Name	D:\Data\user\zhongmei 14-07-07-2.d	Acquisition Date	7/7/2014 3:28:11 PM
Method	tune_wide.m	Operator	BDAL@CN
Sample Name	1	Instrument	micrOTOF-Q II
Comment			

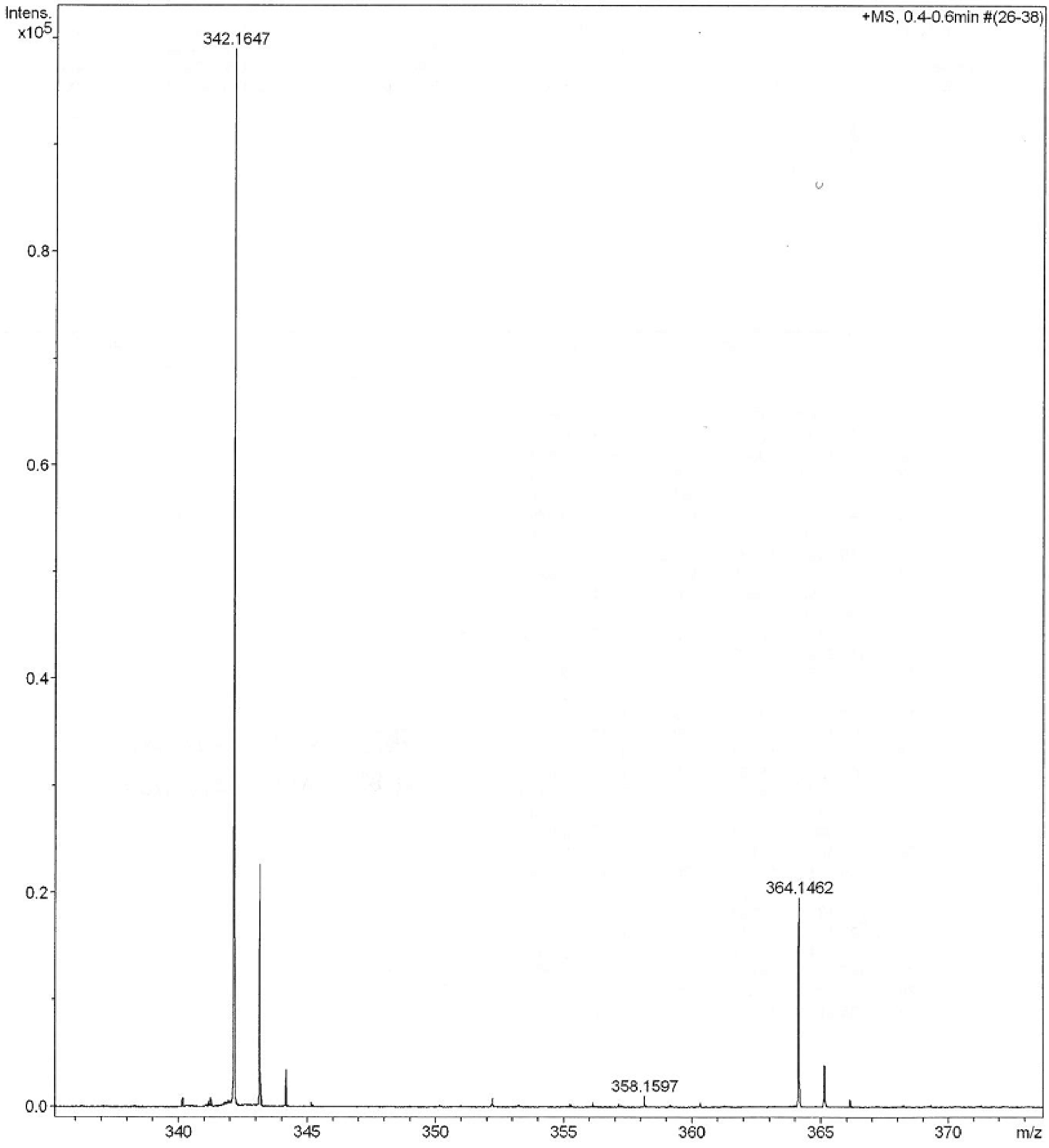

Figure S6. ^{1}H-NMR (400 MHz, $CDCl_3$) spectra of isocorydione (**2**).

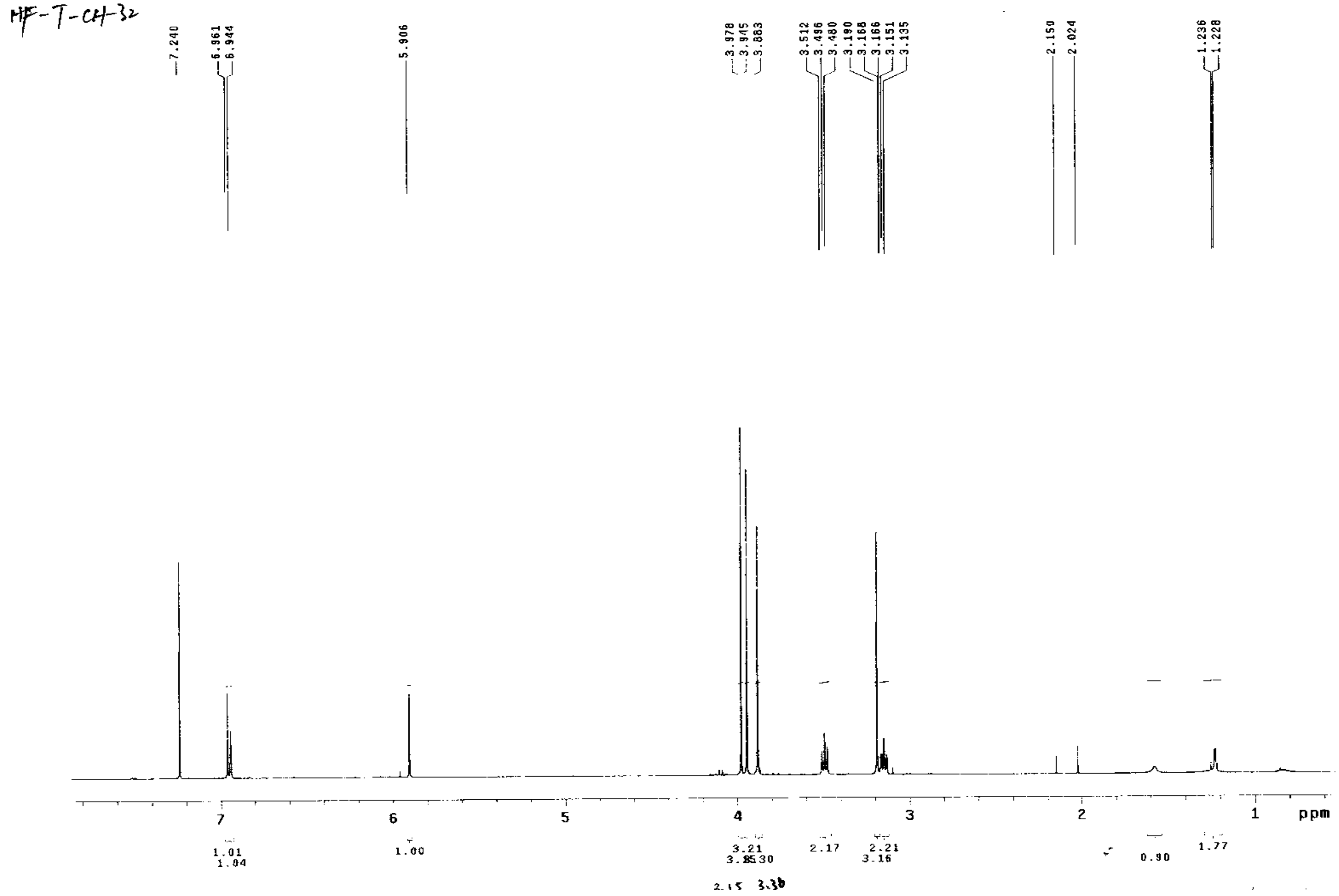

Figure S7. ^{13}C-NMR (100 MHz, $CDCl_3$) spectra of isocorydione (**2**).

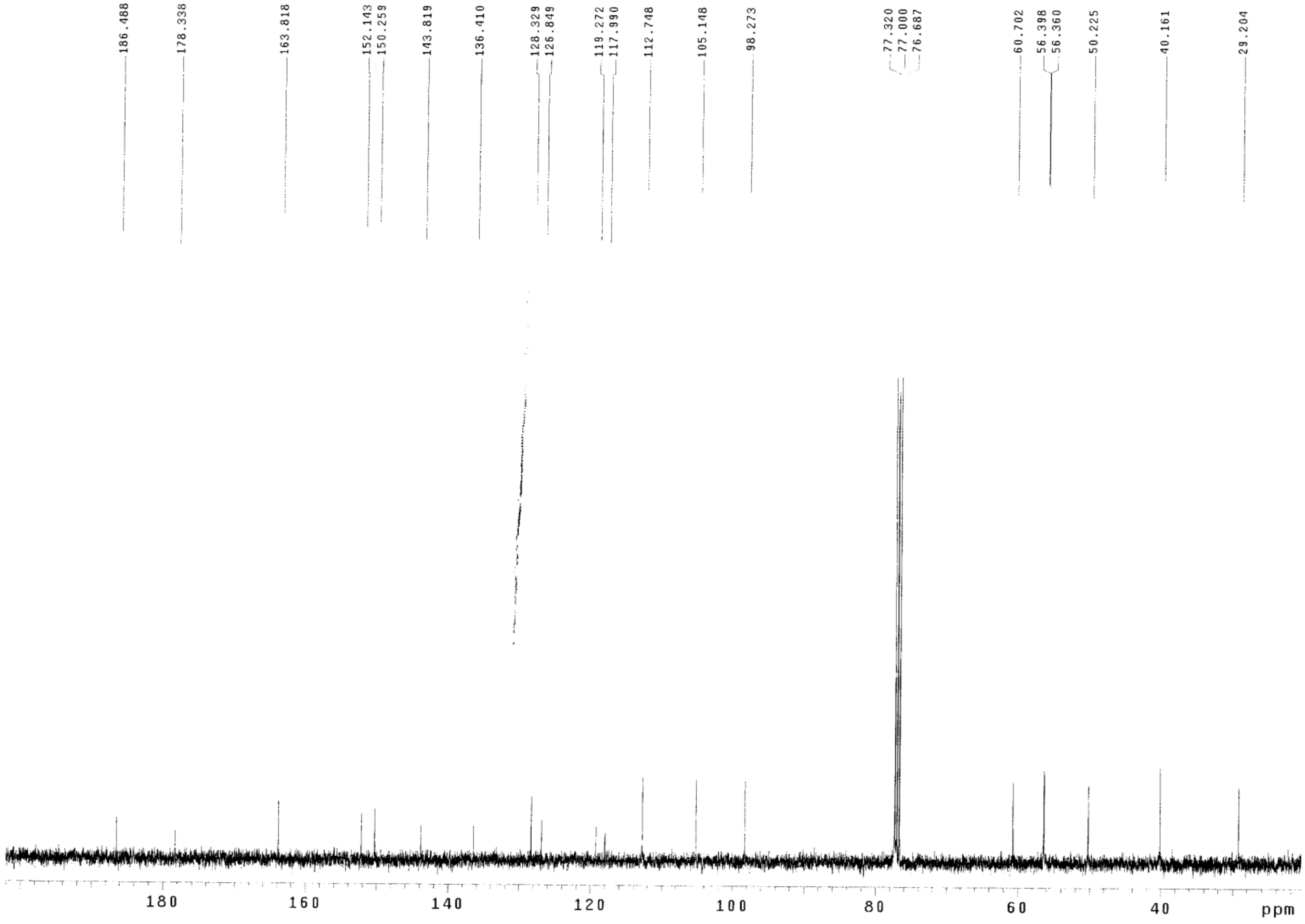

Figure S8. HRMS (ESI) spectra of isocorydione (**2**).

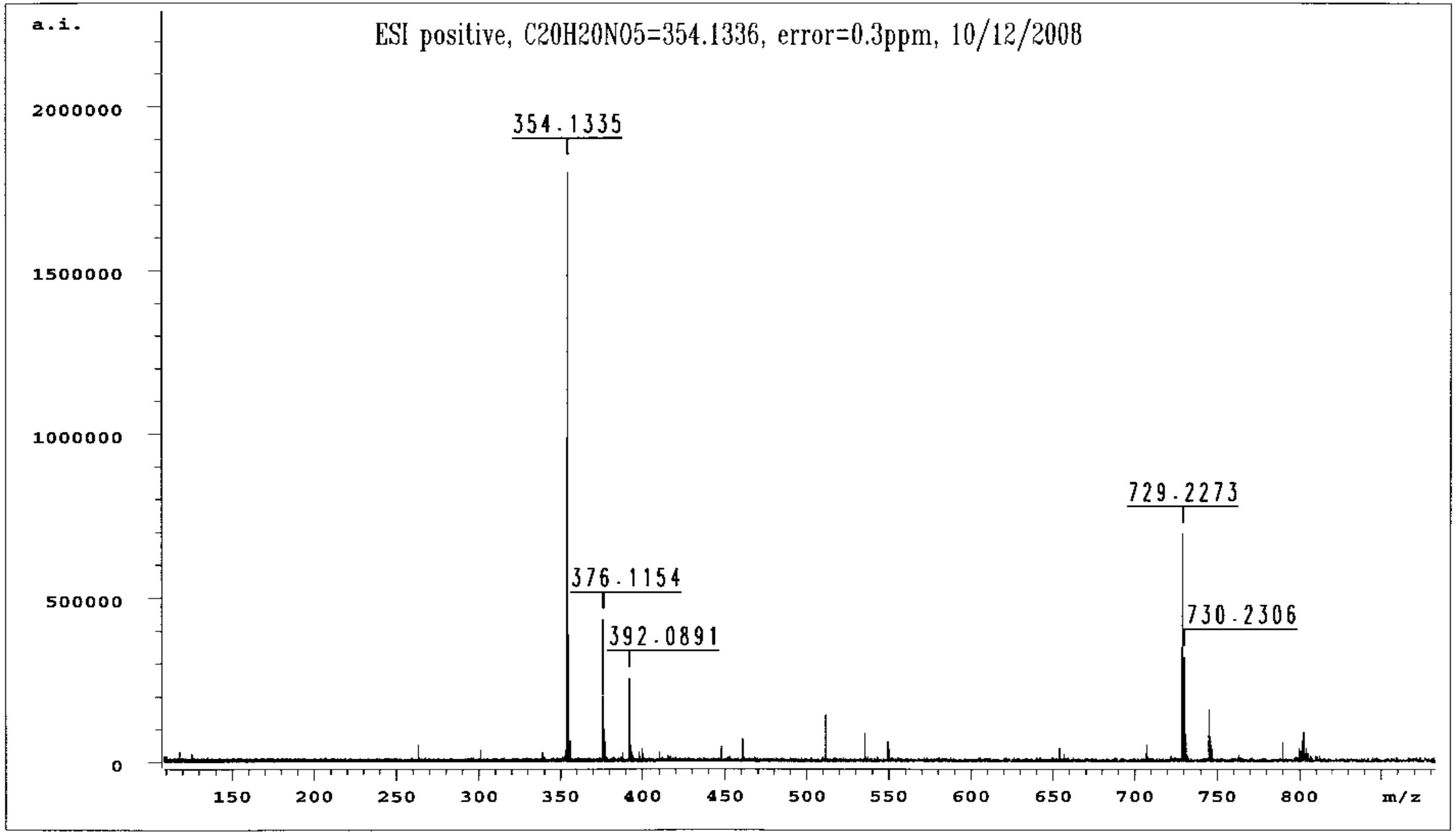

/u/data/TRAINING/gonghongfei1210/1/pdata/1 xspec Wed Dec 10 10:15:35 2008

Figure S9. ^{1}H-NMR (400 MHz, $CDCl_3$) spectra of 4-hydroxy-isocorydione (**3**).

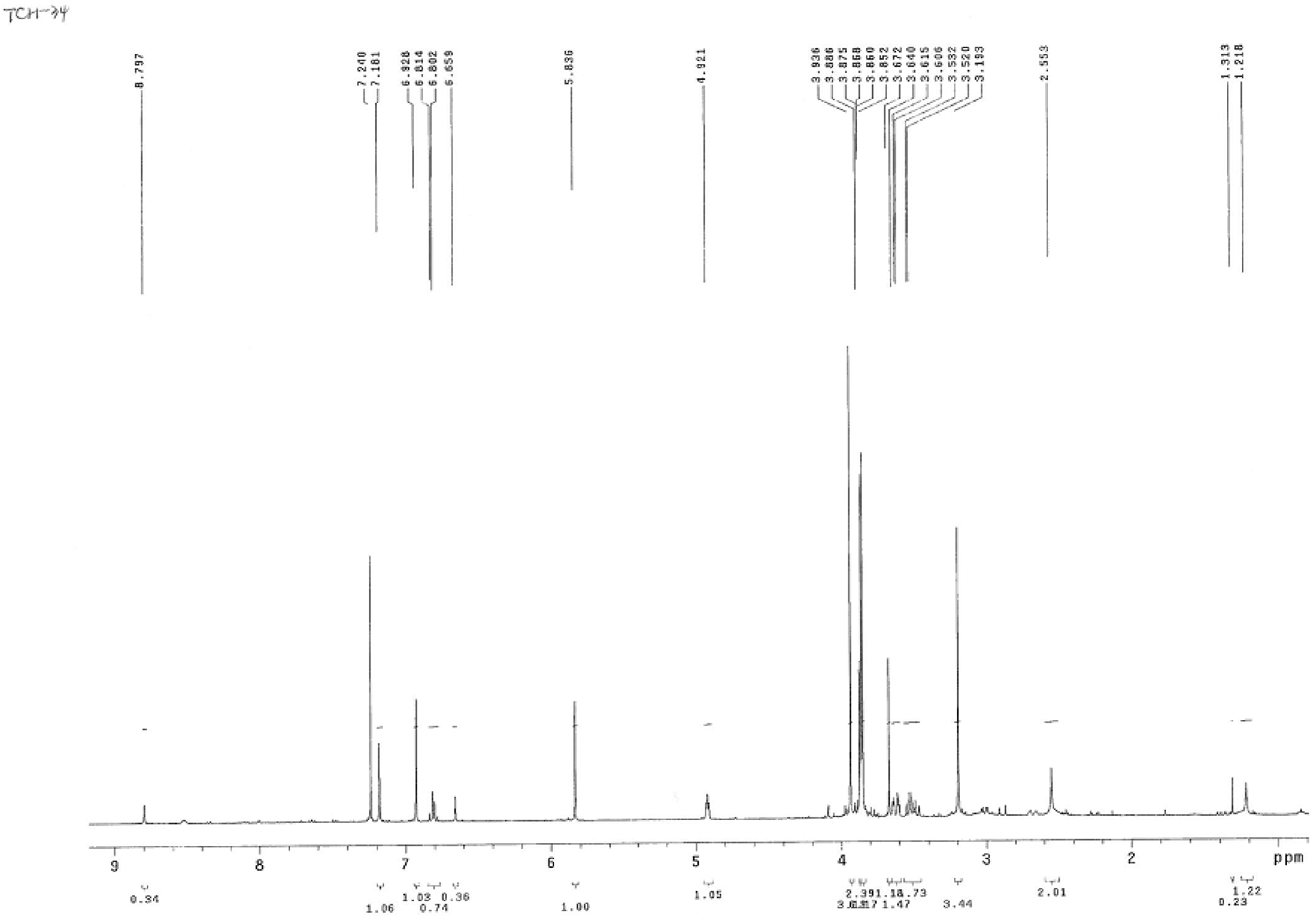

Figure S10. ^{13}C-NMR (100 MHz, $CDCl_3$) spectra of 4-hydroxy-isocorydione (**3**).

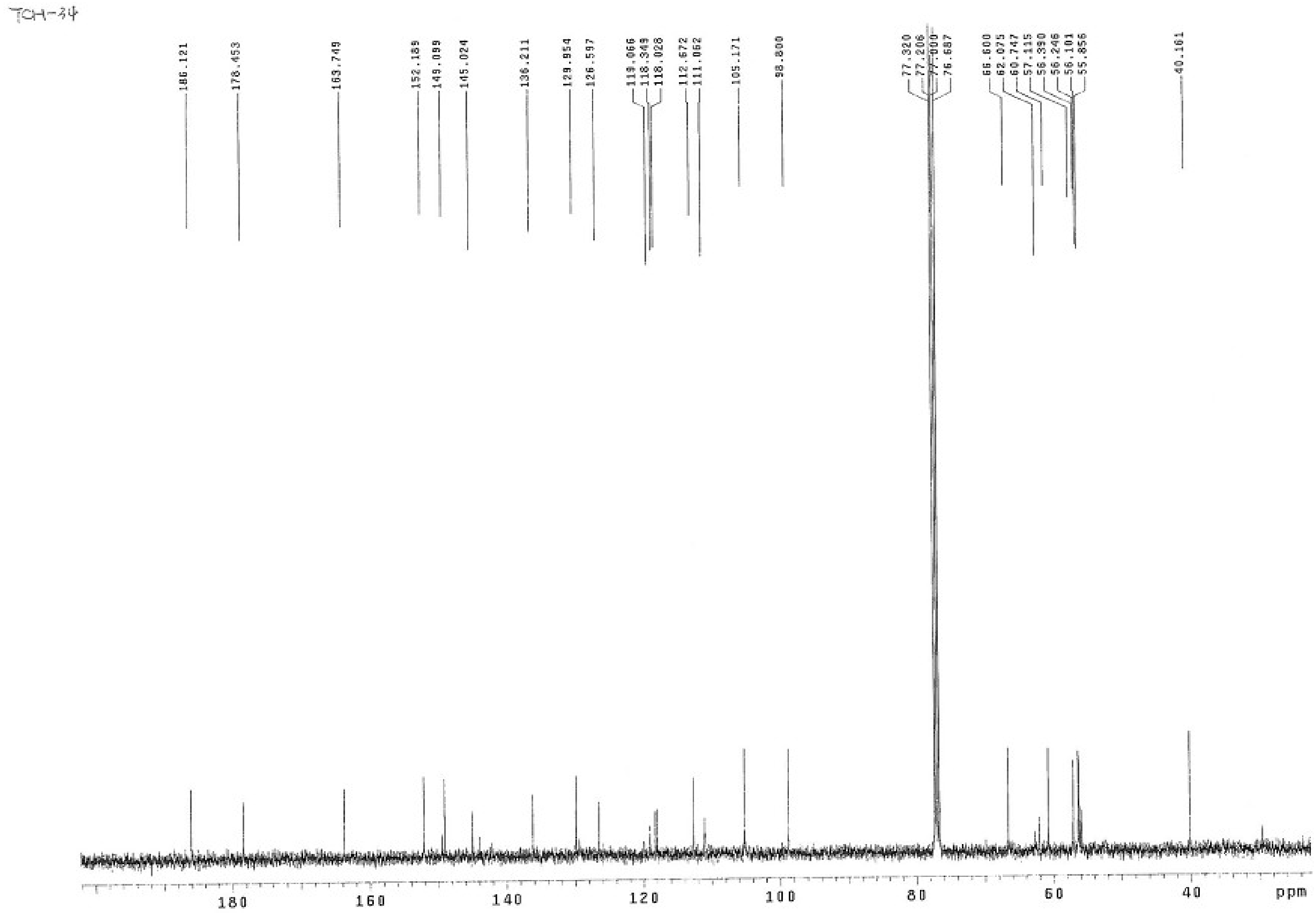

Figure S11. HRMS (ESI) spectra of 4-hydroxy-isocorydione (**3**).

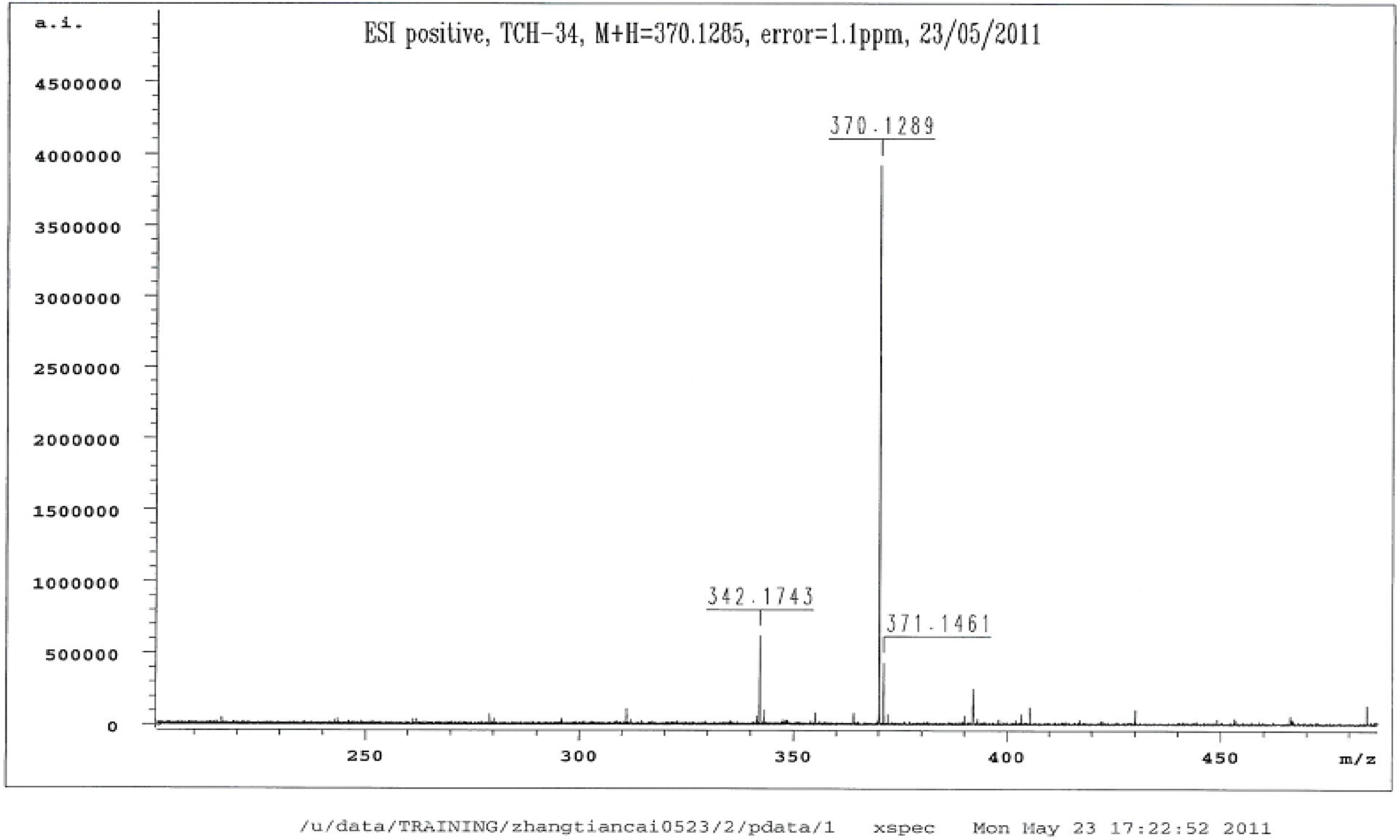

Figure S12. ^{1}H-NMR (400 MHz, $CDCl_3$) spectra of N-demethyl-isocorydione (**4**).

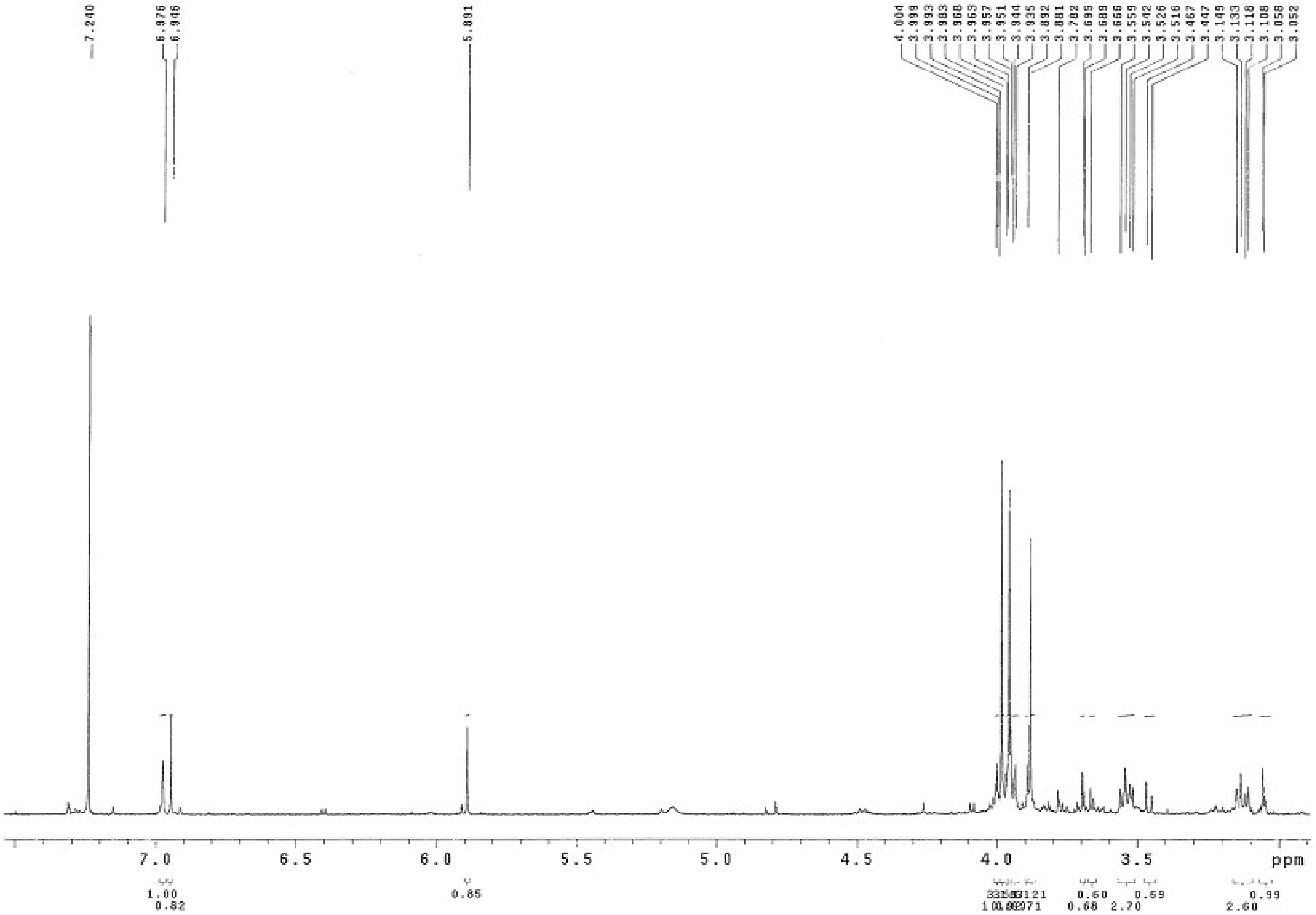

Figure S13. ^{1}H-NMR (400 MHz, $CDCl_3$) spectra of N-demethyl-isocorydione (**4**).

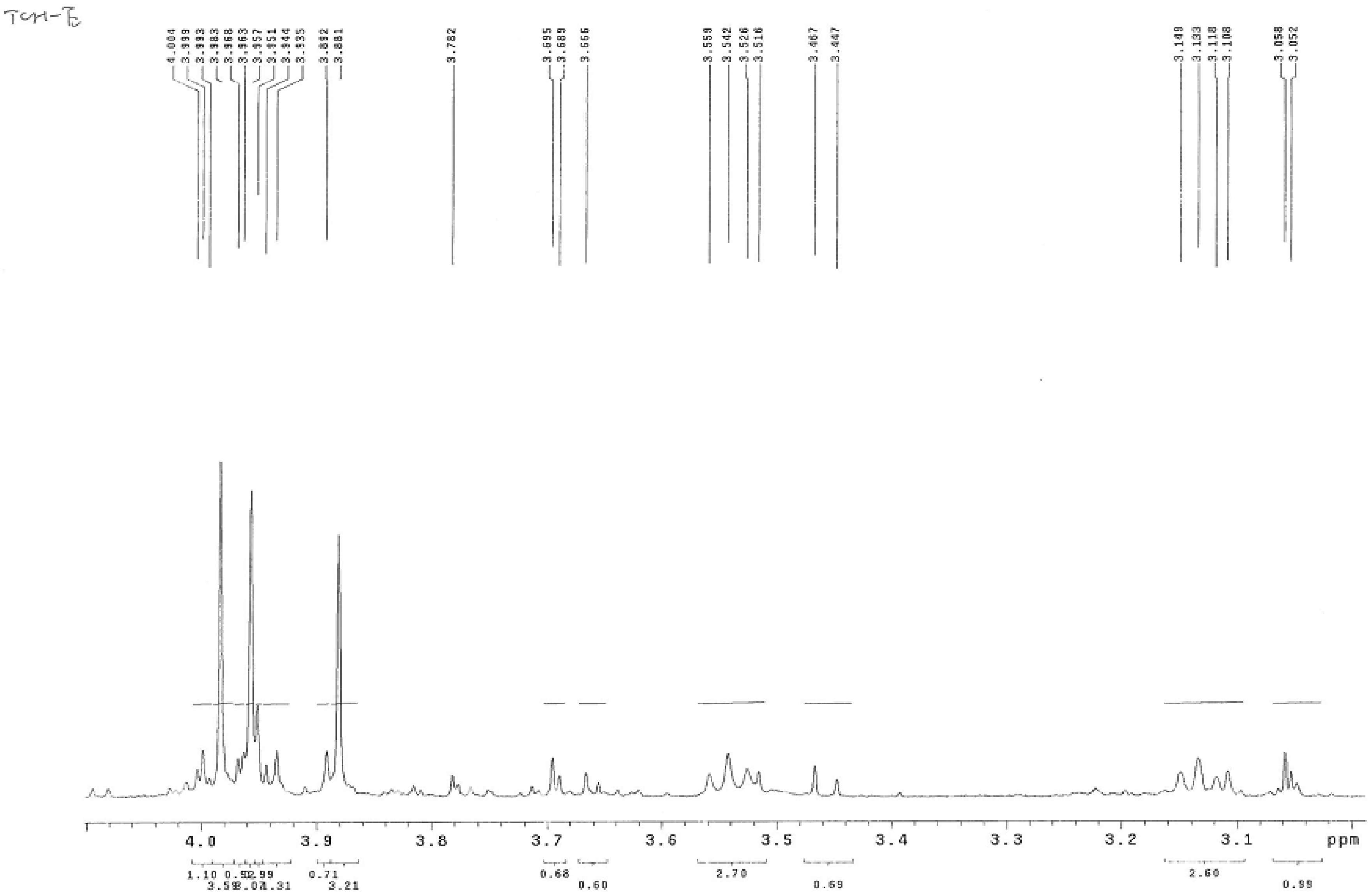

Figure S14. ^{13}C-NMR (100 MHz, $CDCl_3$) spectra of N-demethyl-isocorydione (**4**).

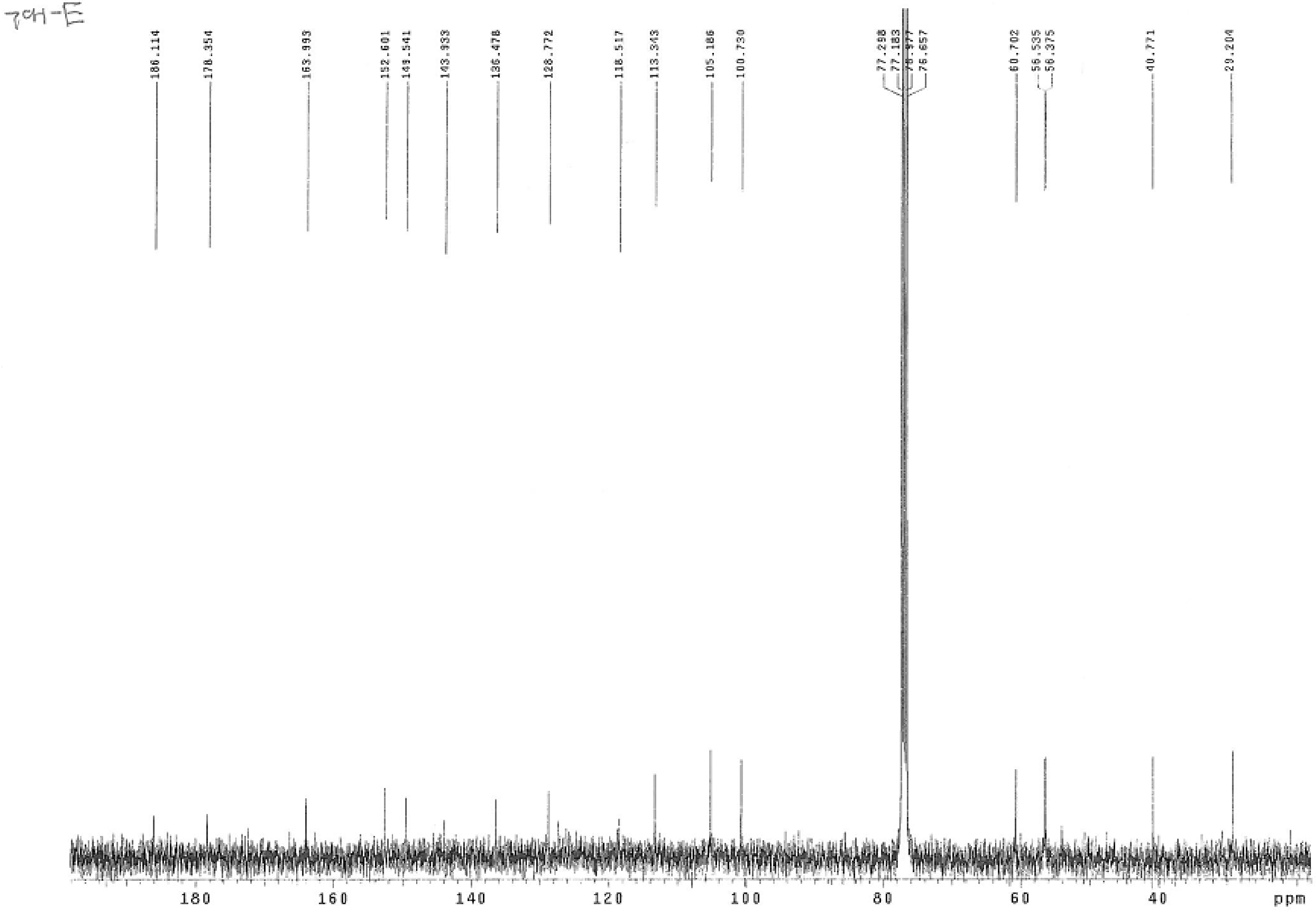

Figure S15. HRMS (ESI) spectra of N-demethyl-isocorydione (**4**).

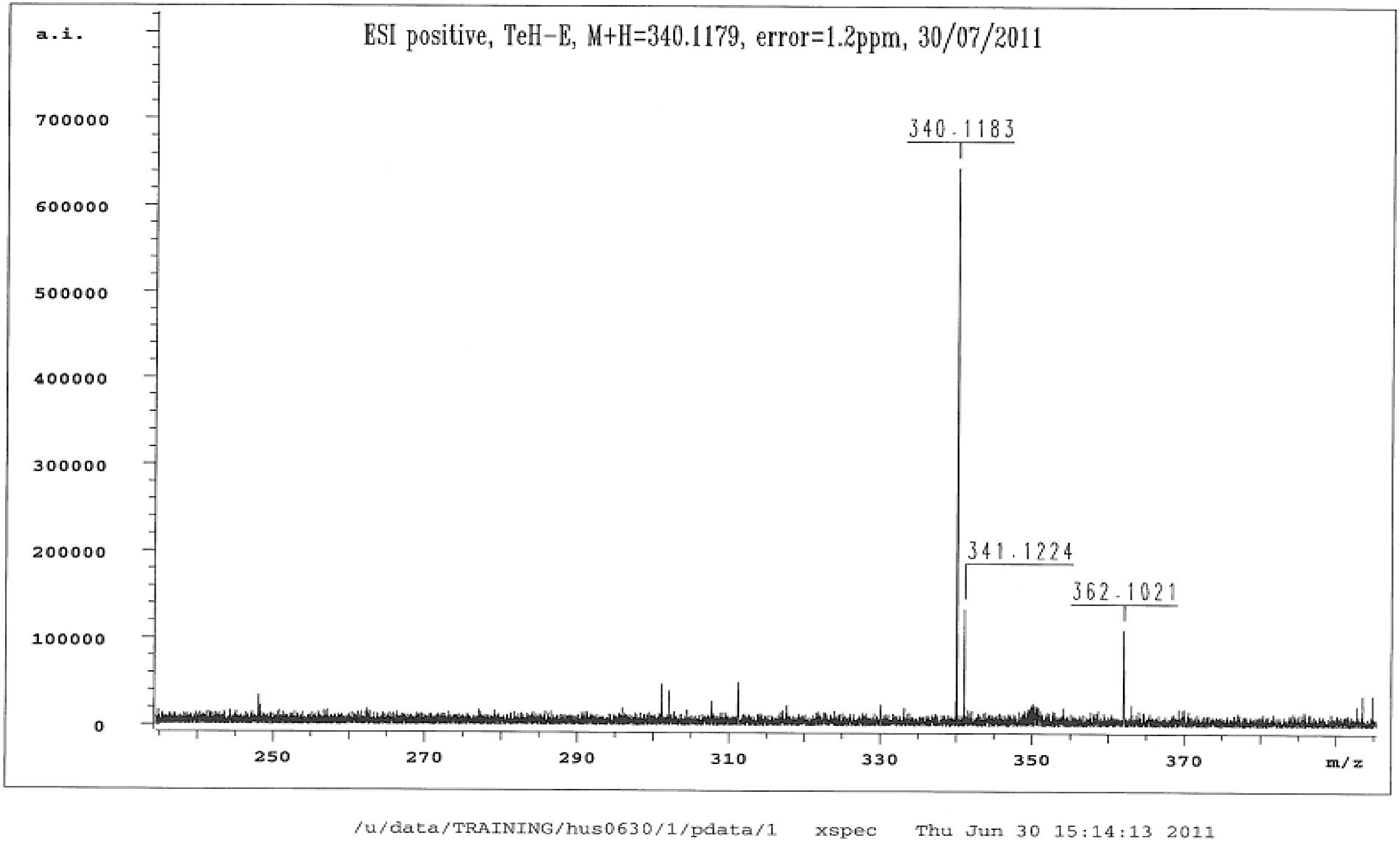

Figure S16. ^{1}H-NMR (400 MHz, $CDCl_3$) spectra of 7-hydroxy-N-demethyl-isocorydione (**5**).

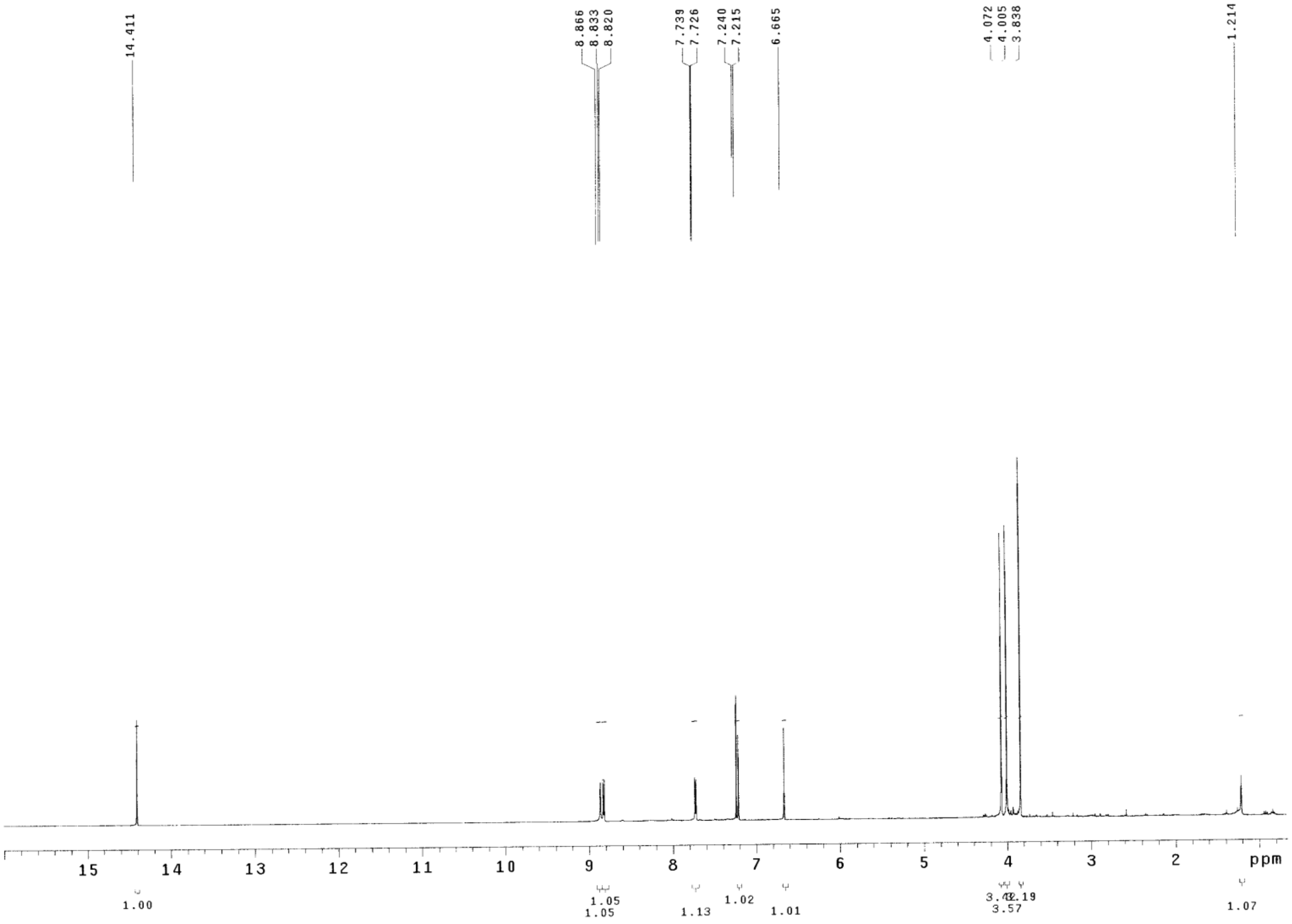

Figure S17. ^{13}C-NMR (100 MHz, $CDCl_3$) spectra of 7-hydroxy-N-demethyl-isocorydione (**5**).

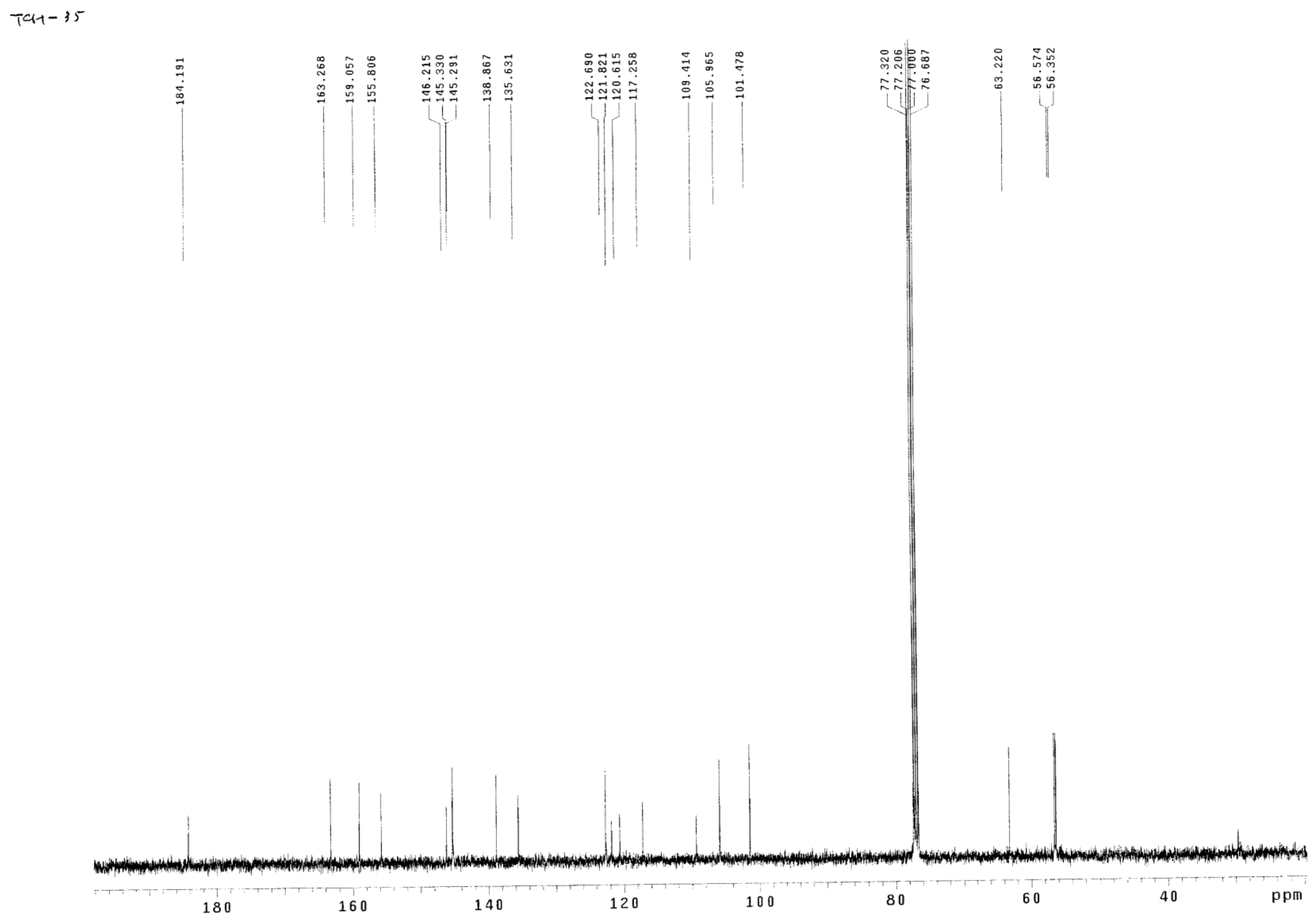

Figure S18. HRMS (ESI) spectra of 7-hydroxy-N-demethyl-isocorydione (**5**).

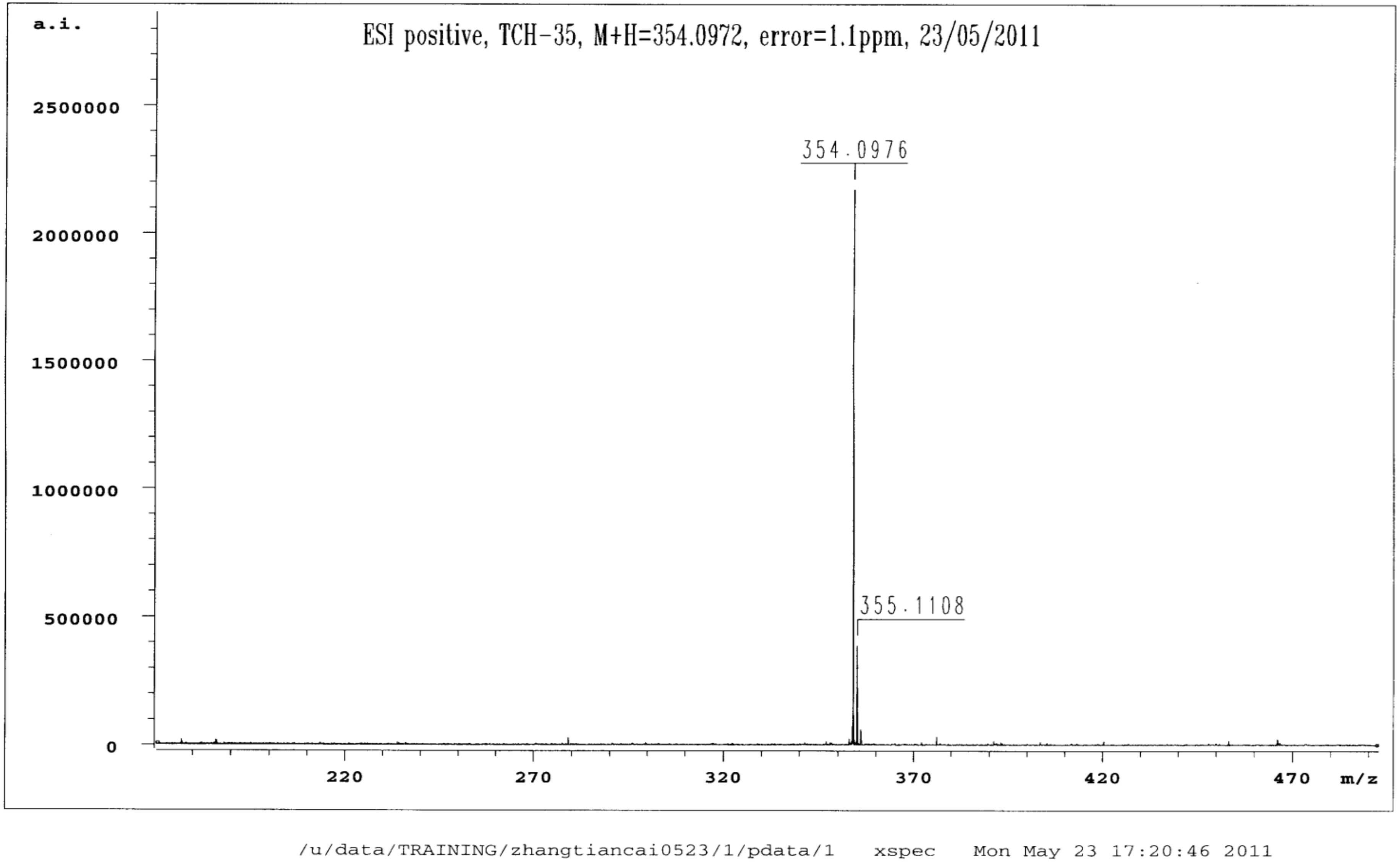

Figure S19. ^{1}H-NMR (400 MHz, $CDCl_3$) spectra of 8-nitro-isocorydine (**7**).

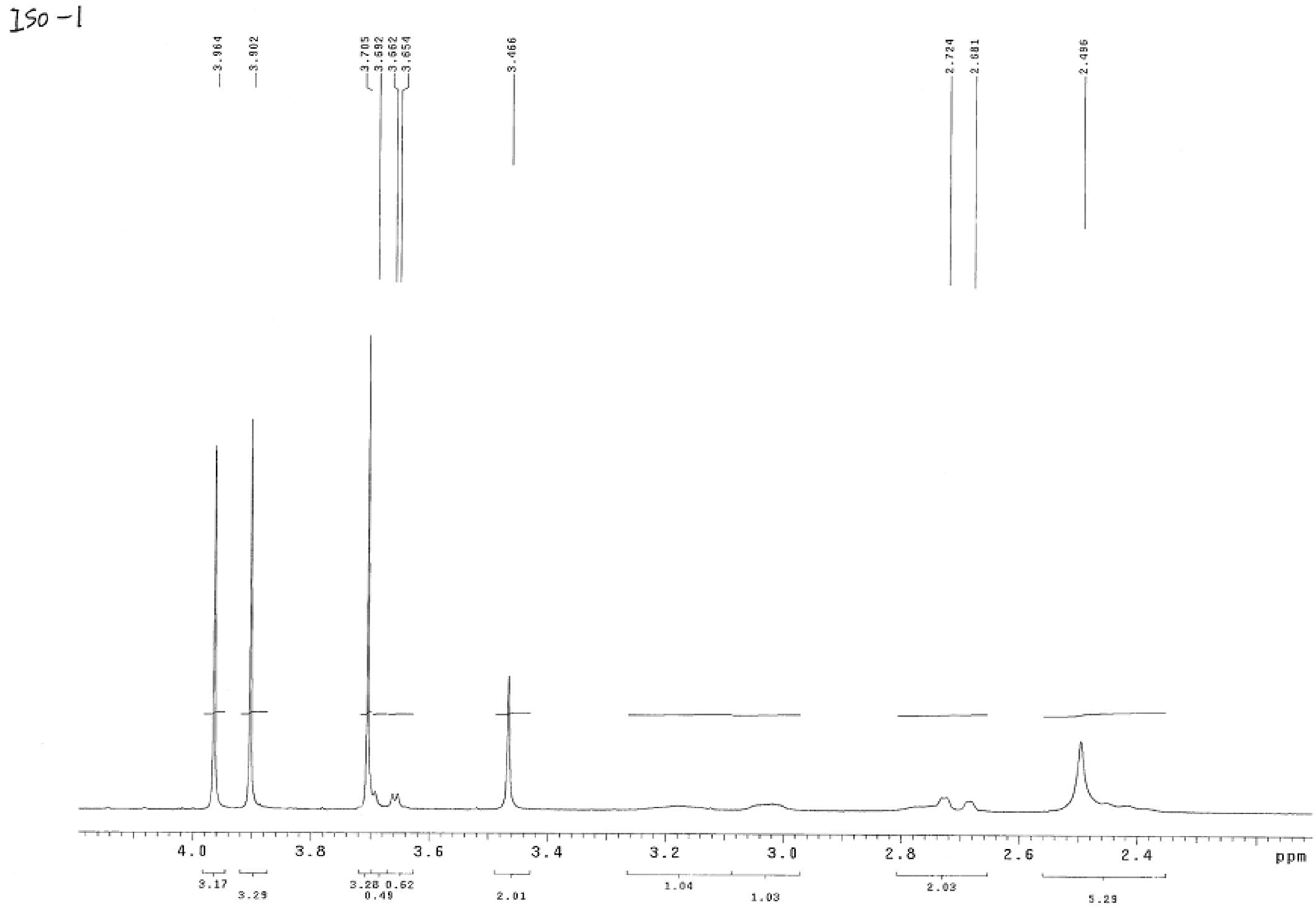

Figure S20. ^{1}H-NMR (400 MHz, $CDCl_3$) spectra of 8-nitro-isocorydine (**7**).

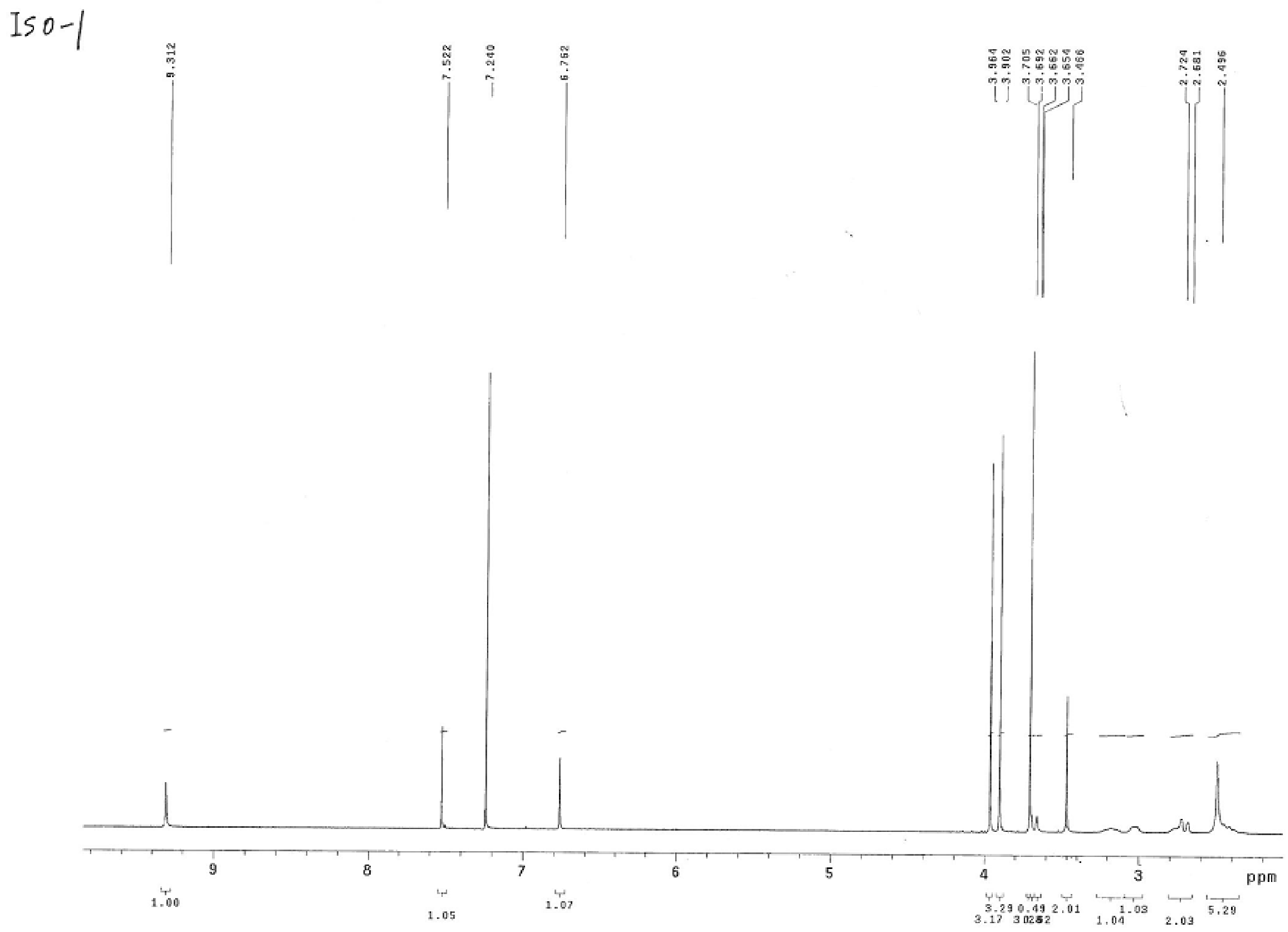

Figure S21. ^{13}C-NMR (100 MHz, $CDCl_3$) spectra of 8-nitro-isocorydine (**7**).

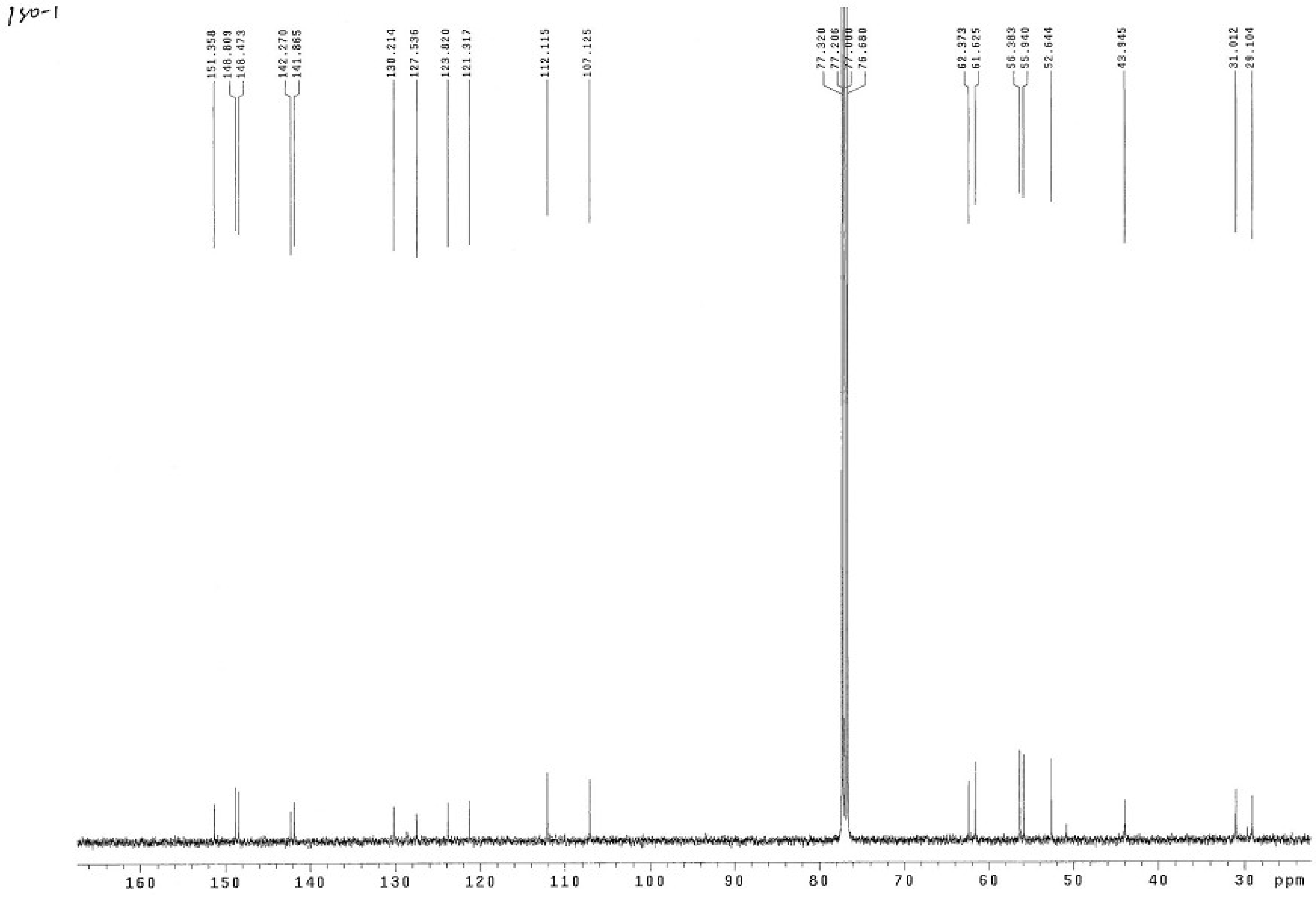

Figure S22. HRMS (ESI) spectra of 8-nitro-isocorydine (**7**).

C8-NO2

Mass Spectrum SmartFormula Report

Analysis Info

Analysis Name D:\Data\User\120628-03.d
Method tune_low.m
Sample Name XQ-2 +
Comment
Operator Hu
Instrument / Ser# micrOTOF-Q II 10241

Acquisition Parameter

Source Type	ESI	Ion Polarity	Positive	Set Nebulizer	0.3 Bar
Focus	Not active	Set Capillary	4500 V	Set Dry Heater	180 ¡ãC
Scan Begin	50 m/z	Set End Plate Offset	-500 V	Set Dry Gas	3.0 l/min
Scan End	1500 m/z	Set Collision Cell RF	120.0 Vpp	Set Divert Valve	Source

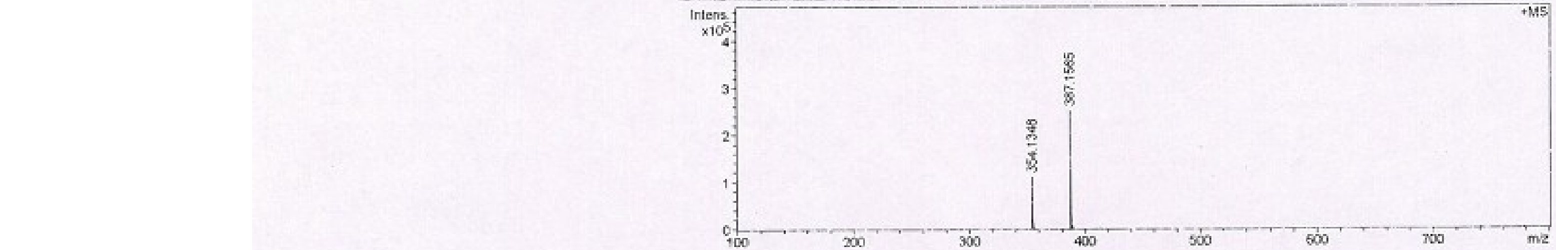

Meas. m/z	#	Formula	Score	m/z	err [ppm]	Mean err [ppm]	mSigma	rdb	e⁻ Conf	N-Rule
354.1348	1	C 20 H 20 N O 5	75.02	354.1336	-3.4	-2.9	0.4	11.5	even	ok
	2	C 19 H 14 N 8	63.34	354.1336	-3.4	-2.9	10.0	17.0	odd	ok
	3	C 21 H 16 N 5 O	100.00	354.1349	0.4	0.9	15.4	16.5	even	ok
	4	C 23 H 18 N 2 O 2	43.08	354.1363	4.1	4.7	21.3	16.0	odd	ok
387.1565	1	C 20 H 23 N 2 O 6	64.77	387.1551	-3.8	-3.0	6.1	10.5	even	ok
	2	C 19 H 17 N 9 O	55.06	387.1551	-3.8	-3.1	14.6	16.0	odd	ok
	3	C 21 H 19 N 6 O 2	100.00	387.1564	-0.3	0.4	20.4	15.5	even	ok
	4	C 23 H 21 N 3 O 3	49.12	387.1577	3.2	3.9	26.8	15.0	odd	ok

Bruker Compass DataAnalysis 4.0 printed: 2012-6-28 11:15:29 Page 1 of 1

Figure S23. ^{1}H-NMR (400 MHz, $CDCl_3$) spectra of 8-amino-isocorydine (**8**).

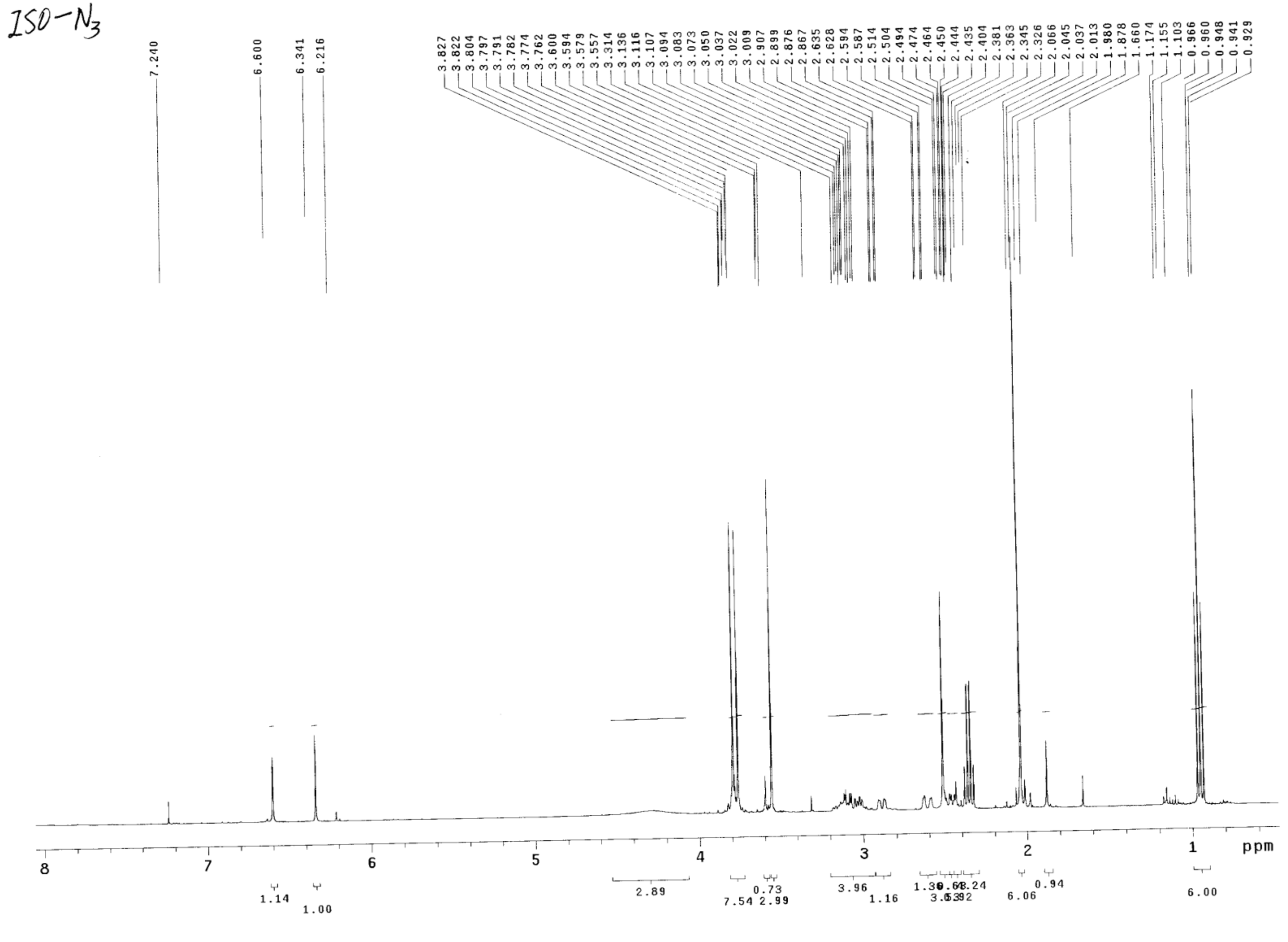

Figure S24. ^{13}C-NMR (100 MHz, $CDCl_3$) spectra of 8-amino-isocorydine (**8**).

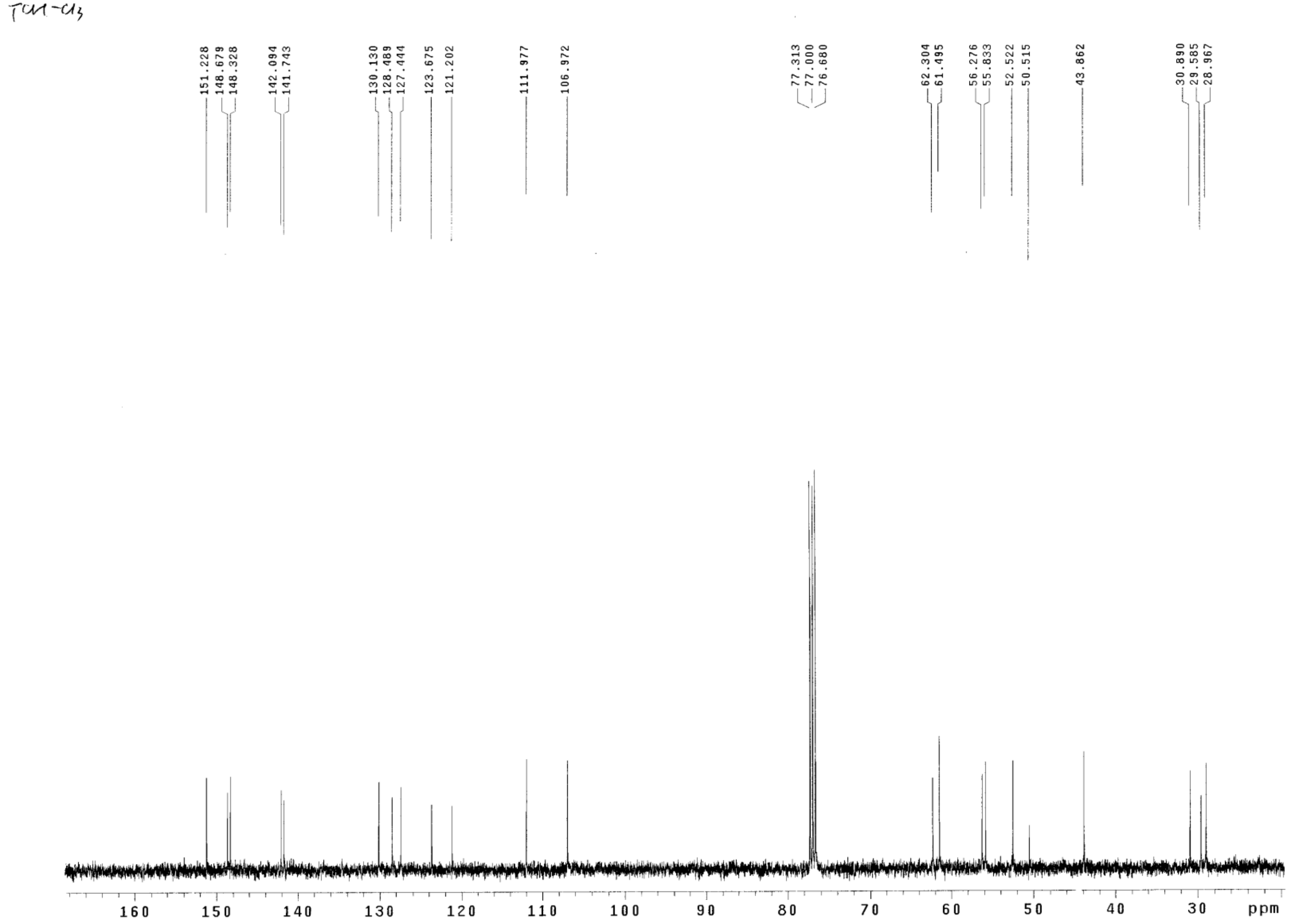

Figure S25. HRMS (ESI) spectra of 8-amino-isocorydine (**8**).

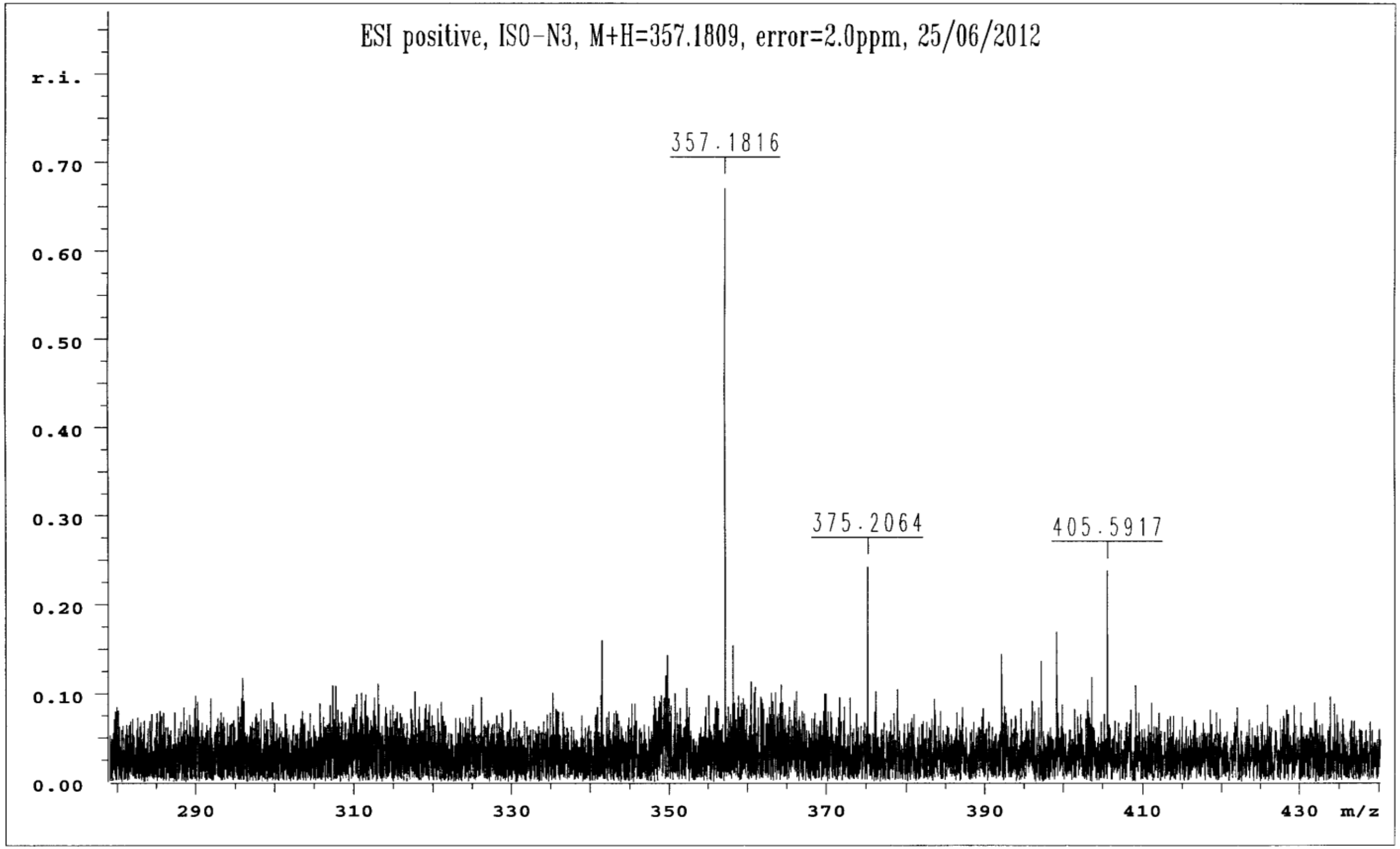

/u/data/TRAINING/liuyanjuan0623/1/pdata/1 xspec Mon Jun 25 16:38:16 2012

Figure S26. ^{1}H-NMR (400 MHz, $CDCl_3$) spectra of 8-chloro-isocorydine (**9**).

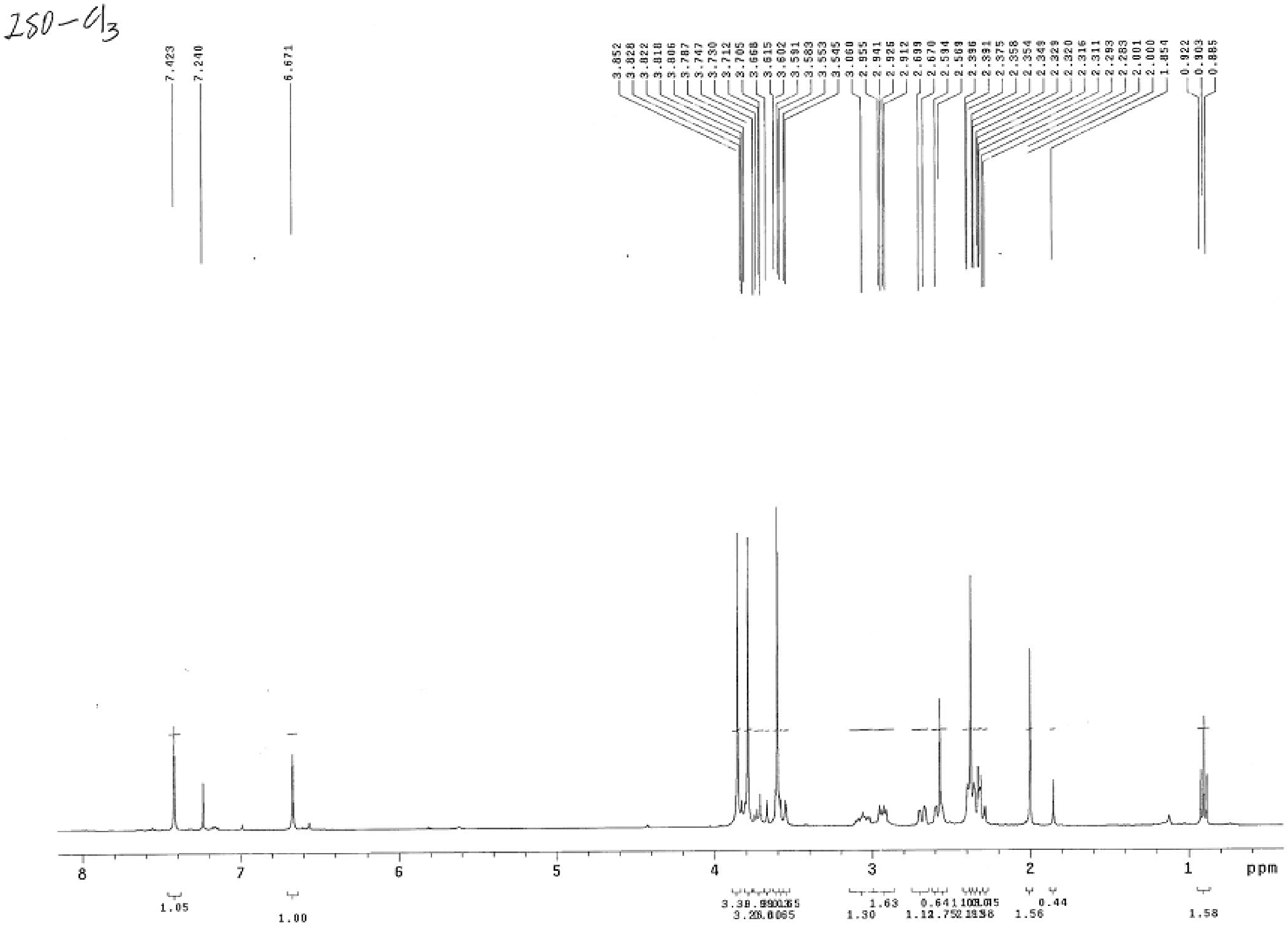

Figure S27. ^{13}C-NMR (100 MHz, $CDCl_3$) spectra of 8-chloro-isocorydine (**9**).

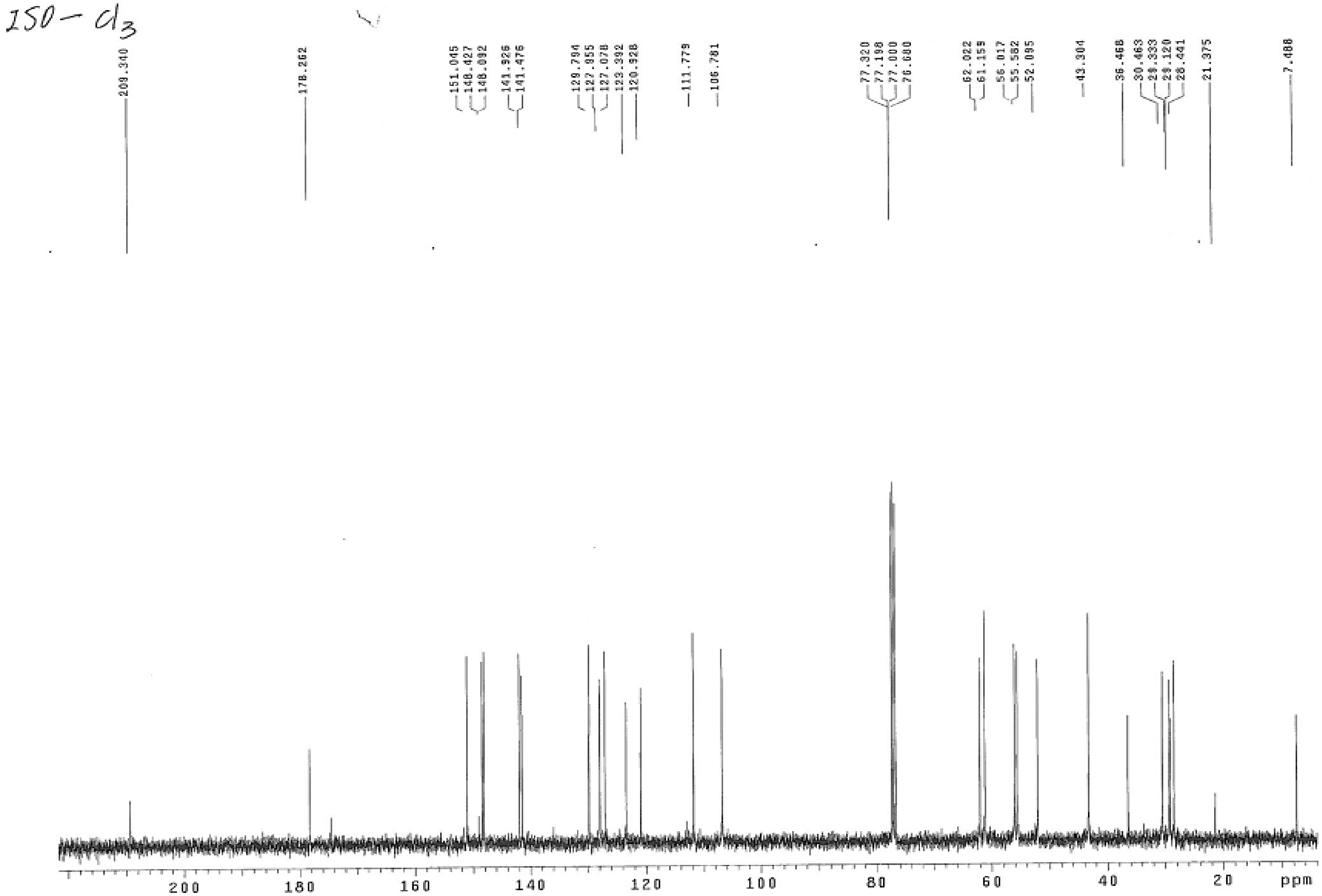

Figure S28. ^{1}H-NMR (400 MHz, $CDCl_3$) spectra of 6a,7-dihydrogen-isocorydione (**10**).

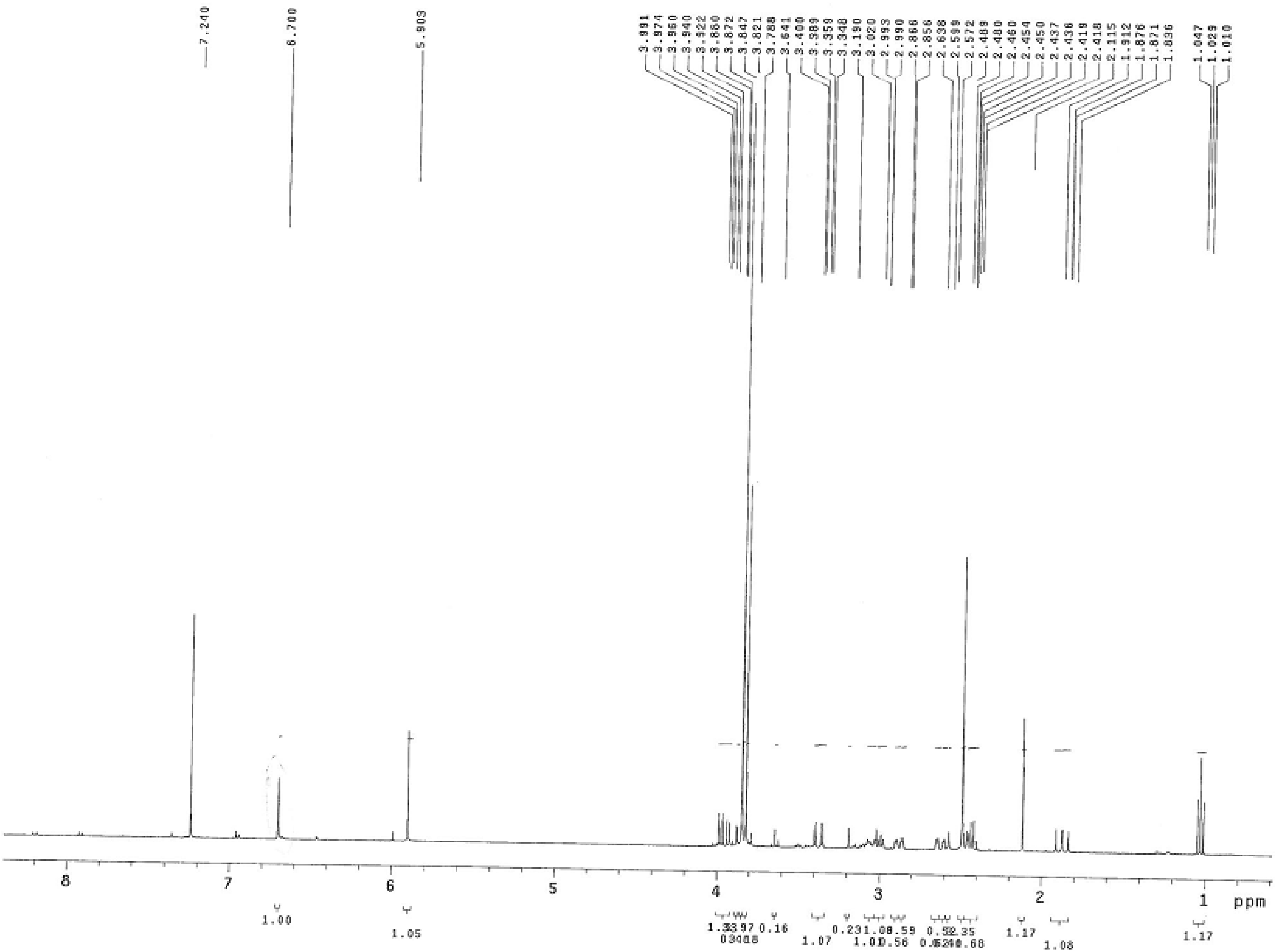

Figure S29. ^{13}C-NMR (100 MHz, $CDCl_3$) spectra of 6a,7-dihydrogen-isocorydione (**10**).

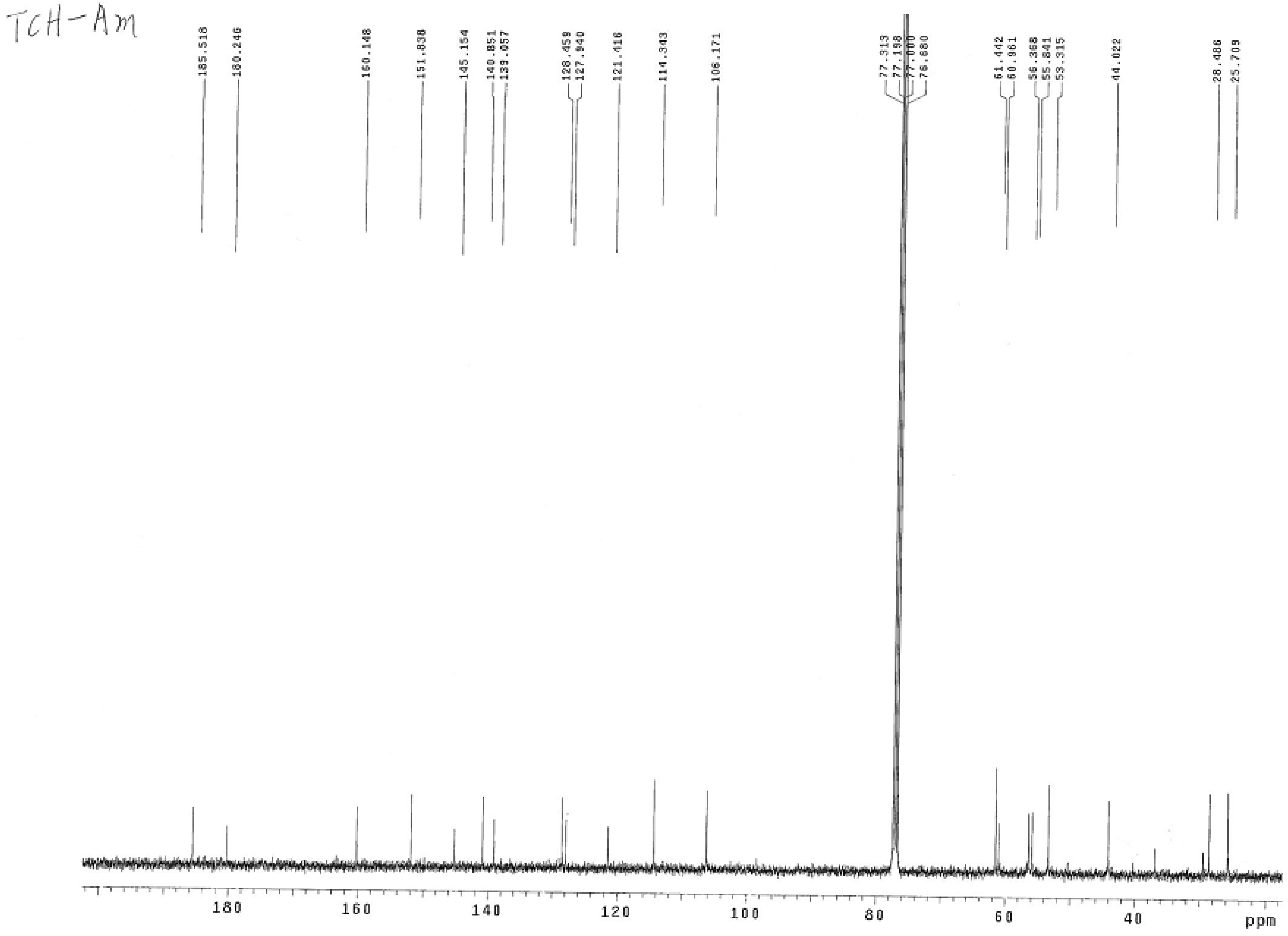

Figure S30. ^{1}H-NMR (400 MHz, $CDCl_3$) spectra of 8-acetamino-isocorydine (**11**).

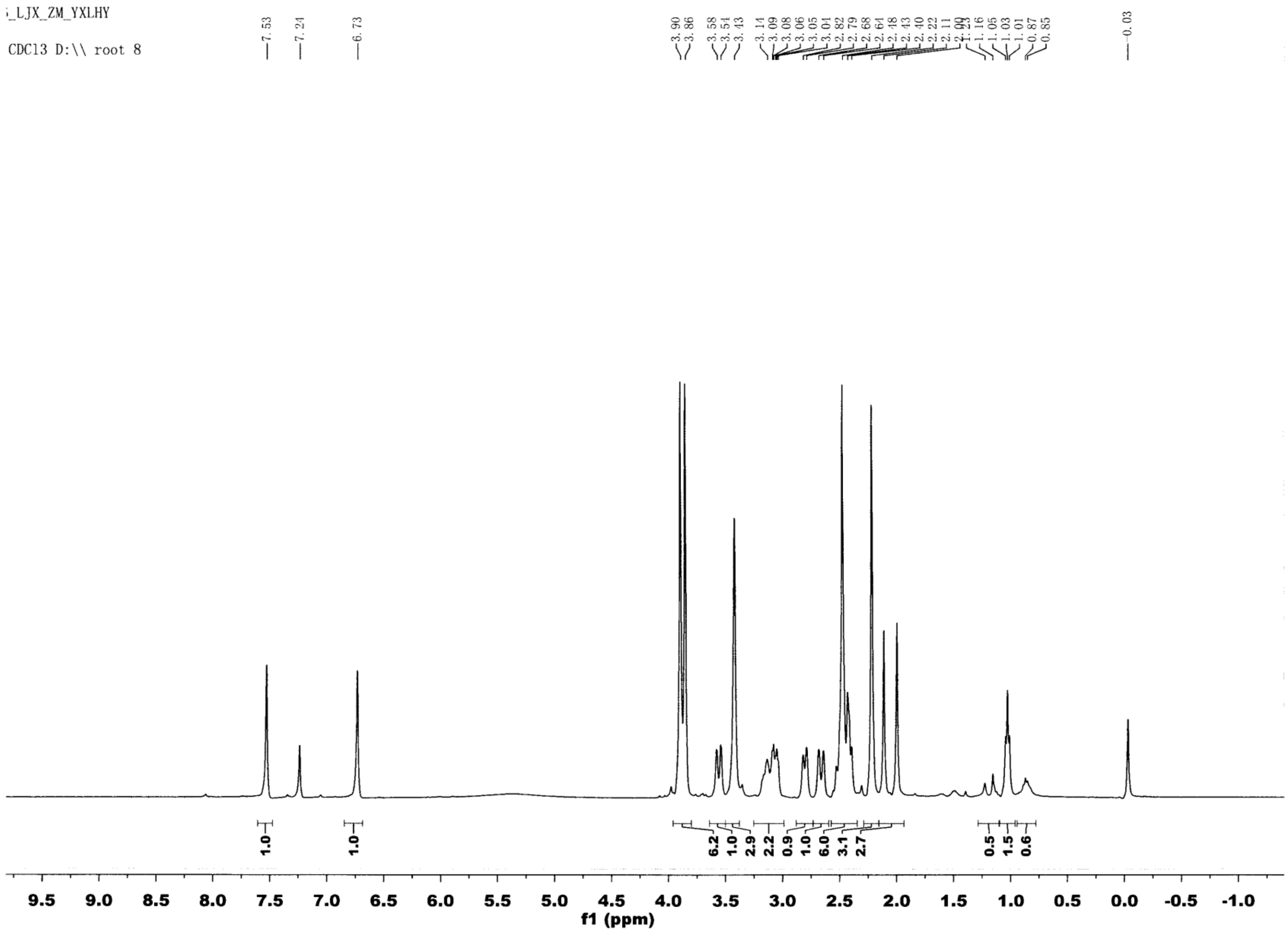

Figure S31. ^{13}C-NMR (100 MHz, $CDCl_3$) spectra of 8-acetamino-isocorydine (**11**).

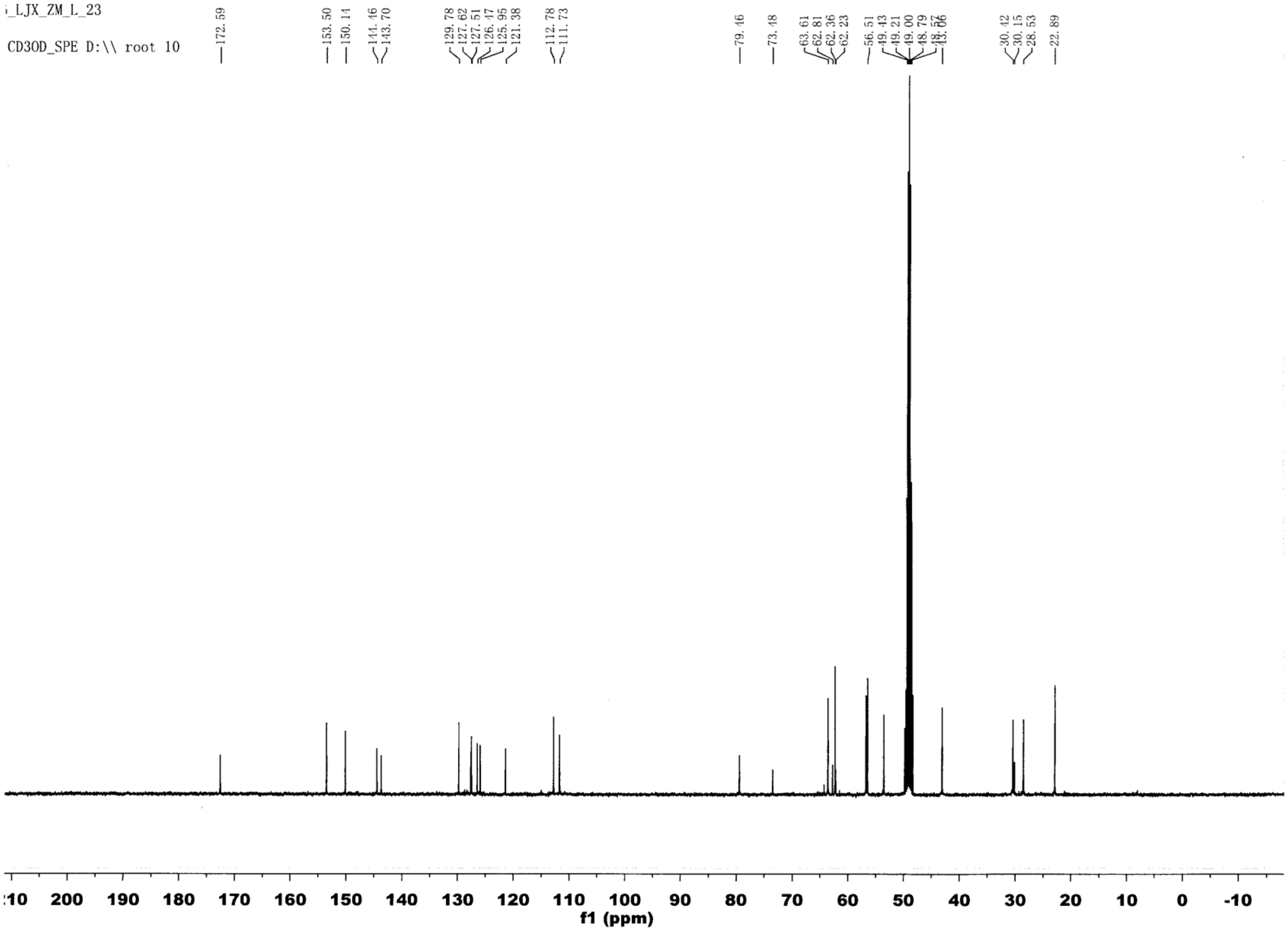

Figure S32. ^{13}C-NMR (100 MHz, $CDCl_3$) spectra of 8-acetamino-isocorydine (**11**).

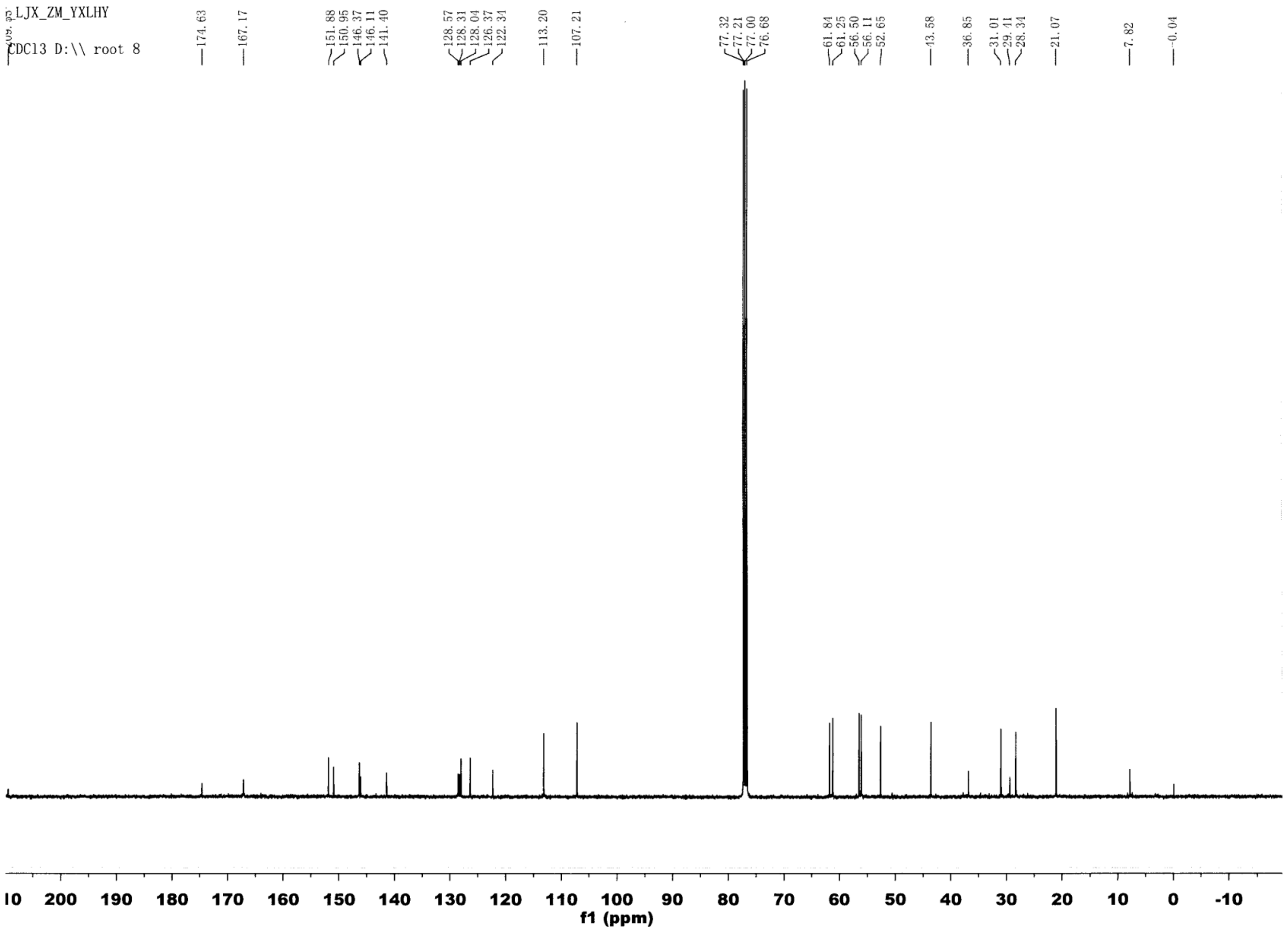

Figure S33. HRMS (ESI) spectra of 8-acetamino-isocorydine (**11**).

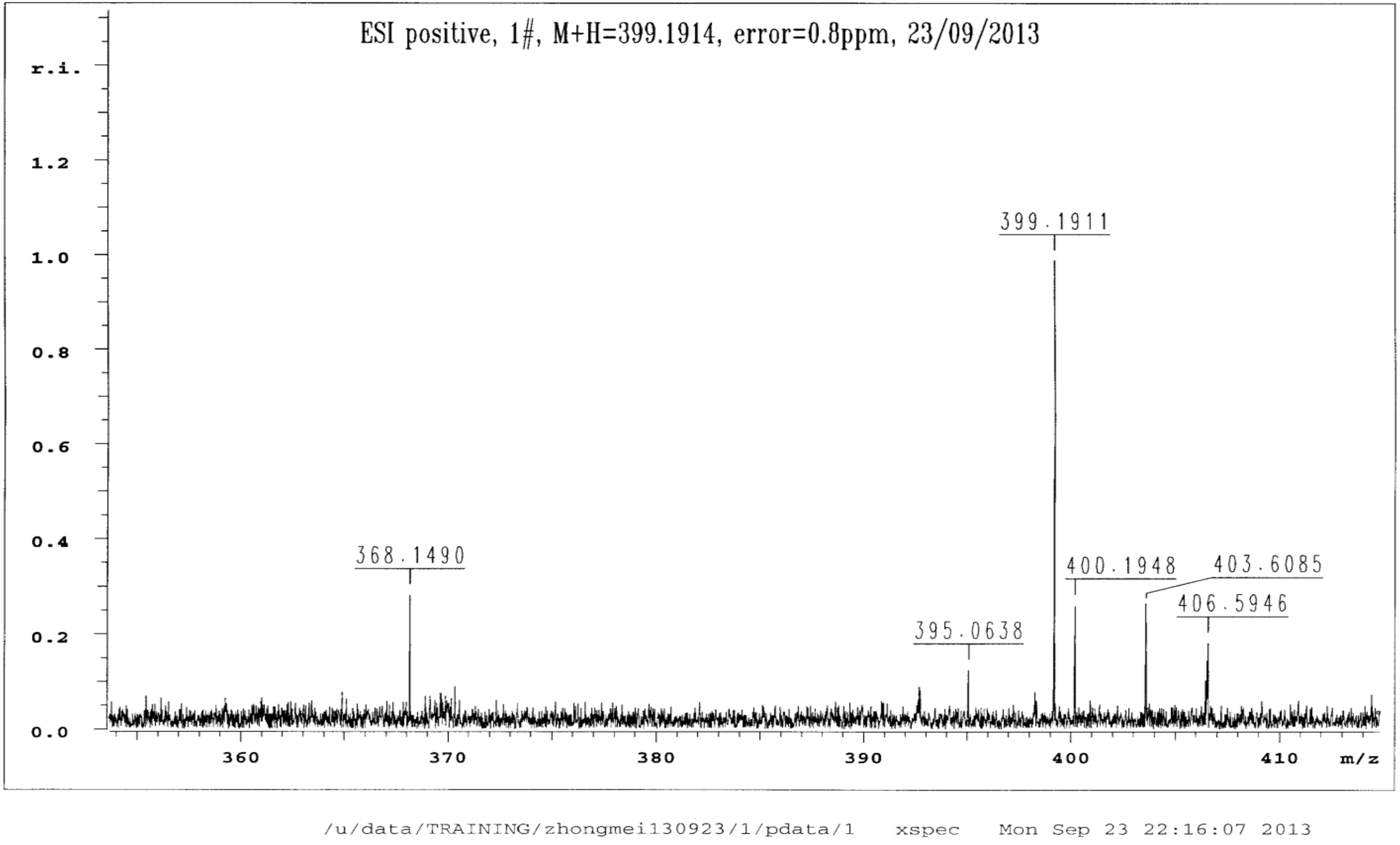

Supplement: Supplementary File 1 [file molecules-19-12099-s001.pdf]
